# Nature-inspired multi-objective artificial intelligence for short-horizon volatility-regime early warning and defensive asset allocation

**DOI:** 10.3389/frai.2026.1953907

**Published:** 2026-09-14

**Authors:** Hamza Boukeffa, Selman Djeffal, Abdelhamid Ghoul

**Affiliations:** 1 Larbi Ben M'hidi University, Oum El Bouaghi, Algeria; 2 National Polytechnic School of Constantine, Constantine, Algeria; 3 Automatic Control, Department of Computer Science, Electrical and Space Engineering, Luleå University of Technology, Luleå, Sweden

**Keywords:** artificial intelligence in finance, defensive asset allocation, metaheuristics, multi-objective optimization, probability calibration, reproducible research, VIX, volatility-regime prediction

## Abstract

Early warning of market turbulence is usually treated as a classification problem, yet a usable system must also produce trustworthy probabilities, stay parsimonious and convert forecasts into defensible positions. We formulate that joint problem as a seven-objective optimisation task in which indicator selection, ridge-logistic regularization, decision threshold, calibration temperature and five defensive-allocation controls are tuned together, and we compare eleven metaheuristics under identical budgets on daily SPY and VIX data from January 2015 to February 2020. The target is a short-horizon volatility-regime exceedance: whether the VIX rises above its rolling 80th percentile within five trading days. Each experiment was repeated over 30 runs and benchmarked against a random-search control, nine conventional strategies, five rolling origins and five alternative stress definitions, with Friedman tests, Holm-corrected pairwise comparisons, effect sizes and bootstrap intervals. In the validation objective space the metaheuristics separate clearly: gray wolf optimisation obtains the best aggregate rank, ant colony optimisation the largest hypervolume and the lowest scalar loss, and most methods dominate random search with large effect sizes. On the untouched holdout the separation disappears. Balanced accuracy lies between 0.66 and 0.70 for every method, run-to-run dispersion equals the between-method spread, no metaheuristic beats random search on any criterion, and stronger validation search is associated with slightly weaker generalization. A ridge-logistic model with a fixed 0.50 threshold matches or exceeds every optimized configuration, and ranking the same days by the current VIX alone reaches an area under the curve of 0.772. The framework performs better under realized-volatility, drawdown and illiquidity labels. Pareto quality and decision quality therefore have to be reported separately. A MATLAB implementation reproducing every result is provided.

## 1 Introduction

Financial stress is a transition in market functioning rather than a single extreme observation. It is reflected in abrupt volatility, weakened liquidity, unstable participation, changing dependence structures, and a rapid deterioration in risk appetite. Fleming et al. (1995) showed that option-implied volatility contains information about subsequent stock-market volatility. Whaley (2000) established the practical role of the VIX as an investor fear gauge and a concise signal of expected market uncertainty. These properties make the VIX suitable for defining an operational early-warning target, but they do not by themselves solve the decision problem faced by a risk manager.

The scope of this study is restricted by design. The target is a short-horizon volatility-regime exceedance: whether the VIX will move above a rolling historical percentile of its own distribution within the next five trading days. That is one operational proxy for market stress, and it is not equivalent to a broad financial-stress index built from credit spreads, funding conditions and cross-market contagion. We therefore make no claim about financial stress in general, and Section 4.6 reports how the same framework behaves when the label is replaced by a realized-volatility spike, a drawdown deepening, an illiquidity spike or a composite stress index, and when every VIX-derived predictor is removed from the candidate set.

Recent artificial-intelligence research has substantially improved financial prediction. Gunnarsson et al. (2024) documented the growing use of machine learning for realized and implied volatility forecasting, while identifying robustness, calibration, and reproducibility as persistent limitations. Campisi et al. (2024) found that volatility-index information can improve machine-learning forecasts of US equity-market direction. Del Fava et al. (2024) demonstrated that international financial stress is forecastable and that the usefulness of additional risk variables varies with the forecasting horizon. Wang and Zhou (2024) showed that nonlinear learning combined with SHAP-based analysis can recover economically meaningful systemic-risk episodes. de Moraes Souza et al. (2024) found that financial stress is driven by several complementary variables, which supports multivariate rather than single-indicator monitoring.

The recent literature in *Frontiers in Artificial Intelligence* further confirms the importance of reliable and application-oriented financial AI. Lokanan and Ramzan (2024) reported strong machine-learning performance for firm-level financial-distress prediction and emphasized stable feature selection. Sutiene et al. (2024) reviewed AI-based portfolio management and concluded that forecasting, portfolio construction, execution, evaluation, and transparency should be considered as connected stages rather than isolated tasks. Agosto et al. (2024) used statistical learning to measure financial fragility under the COVID-19 shock, illustrating the need to account for changing economic regimes. Cao and Geman (2025) showed that probability adjustment can materially improve return and volatility forecasting, highlighting the value of probability quality rather than classification alone. Bussmann et al. (2025) demonstrated that parsimonious and explainable financial predictors can preserve useful risk information while improving accountability. Rohan et al. (2025) reviewed the evolution of AI-driven market prediction and identified integration between forecasting and investment decision-making as a central research direction.

Despite this progress, most studies optimize only one part of the pipeline. Prediction accuracy is often improved without calibrating the resulting probabilities. Feature selection is commonly treated as preprocessing rather than an explicit objective. Portfolio control is frequently evaluated separately from the forecasting model that generates its signal. Random data partitions can also expose training procedures to future market regimes. Finally, a metaheuristic that approximates a Pareto front accurately may still yield an operational solution with weak stress sensitivity or poor economic performance.

Our work addresses these limitations through a unified multi-objective formulation in which the forecast model and defensive allocation policy are optimized together. The contribution is not another hybrid optimizer. The contribution is a rigorous, transparent, and reproducible decision framework that compares eleven established metaheuristics under exactly the same computational and chronological conditions. The framework simultaneously minimizes predictive loss, false-negative risk, calibration loss, feature ratio, temporal instability, maximum drawdown, and financial inefficiency. This design connects statistical learning quality with the consequences of acting on the resulting probabilities.

A second contribution concerns the interpretation of optimizer comparisons. Once the experiment is repeated 30 times, compared against a random-search control and against conventional financial baselines, and tested over several rolling origins, the ordering of the optimizers in the validation objective space proves stable and statistically strong, while the ordering of the same optimizers on untouched data does not. We report both results. On a sample of roughly one thousand trading days, an eight-parameter policy combined with a 28-variable feature gate can be tuned to almost any validation profile, and the resulting differences do not carry over to the following market window.

The principal contributions are:

1. A leakage-controlled short-horizon volatility-regime target that asks whether the VIX will exceed its rolling historical threshold during the following five trading days.
2. A mixed decision vector that jointly optimizes indicator selection, regularization, decision threshold, calibration temperature, and five defensive-allocation controls.
3. A seven-objective formulation that preserves missed-event risk, calibration, parsimony, regime stability, drawdown, and economic efficiency as separate criteria.
4. A repeated-run benchmark of PSO, ABC, GA, CS, BOA, WOA, POA, GWO, TLBO, DE, and ACO using identical evaluation budgets and a common non-dominated archive, extended in this revision to 30 independent runs and to a uniform random-search control with the same budget.
5. A complete statistical treatment: Friedman and Iman–Davenport omnibus tests, Holm-adjusted pairwise Wilcoxon signed-rank tests over all 55 comparisons, rank-biserial and Cliff's δ effect sizes, and bootstrap confidence intervals for every reported difference.
6. A benchmark against nine conventional reference strategies, a five-origin rolling evaluation, five alternative stress definitions and an ablation without any VIX-derived predictor, which together delimit what the framework does and does not demonstrate.
7. A self-contained MATLAB implementation supplied with the paper, allowing readers to reproduce every numerical result and extend the framework to other assets, stress definitions, and classifiers.

The remainder of the paper is organized as follows. Section 2 reviews financial AI, probability calibration, portfolio control, backtest overfitting and the compared metaheuristics. Section 3 defines the data, target, decision vector, seven objectives, defensive policy, reference strategies, rolling-origin design and evaluation protocol. Section 4 reports the predictive, calibration, optimization, robustness and backtest results. Section 5 discusses the implications. Section 6 presents the limitations. Section 7 concludes.

## 2 Related work and optimization background

### 2.1 Financial stress prediction, probability quality, and investment decisions

In Gunnarsson et al. (2024), the authors showed that AI-based volatility forecasting has advanced rapidly, but model comparison is often weakened by inconsistent validation and limited probability assessment. In Campisi et al. (2024), the authors demonstrated that volatility indices can strengthen equity-direction forecasts. In Del Fava et al. (2024), the authors found that international stress forecasting depends on both the information set and the prediction horizon. In Lokanan and Ramzan (2024), the authors reported high firm-level distress-prediction accuracy, whereas our work addresses market-level volatility regimes and explicitly links the forecast to a defensive exposure decision. In Agosto et al. (2024), the authors quantified financial fragility under a major economic shock, while our framework evaluates low-, medium-, and high-VIX regimes inside a chronological holdout design.

In Sutiene et al. (2024), the authors argued that AI portfolio management should connect signal generation, portfolio construction, execution, and evaluation. Our work operationalizes that connection by optimizing the classifier and exposure policy in the same decision vector. In Cao and Geman (2025), the authors improved return and volatility forecasts through a probability adjustment designed for news hype. Our study reaches the same broader conclusion from a different direction: probability quality must be treated as a first-class criterion because the allocation policy consumes probabilities rather than hard class labels. In Bussmann et al. (2025), the authors used explainable learning to identify parsimonious risk drivers. Our framework similarly rewards compact indicator sets, but it additionally requires temporal stability and economic utility. In Rohan et al. (2025), the authors reviewed AI methods for market prediction and emphasized the increasing integration of predictive systems with portfolio decisions. Our contribution provides a controlled multi-objective benchmark for that integration.

Calibration is essential when probabilities trigger financial actions. In Guo et al. (2017), the authors showed that strong discrimination can coexist with unreliable confidence estimates. In Brier (1950), a proper quadratic score was introduced for probabilistic forecasts. Our work therefore evaluates both the Brier score and expected calibration error and jointly optimizes a temperature-scaling coefficient. Liquidity is represented through the Amihud proxy introduced in Amihud (2002), which connects absolute return variation with traded value.

### 2.2 Backtest overfitting and the role of simple baselines

A search procedure that repeatedly evaluates trading rules on one historical window will eventually find a rule that looks profitable by chance. White formalized this problem and proposed a reality check for data snooping White, (2000). Bailey et al. (2014) showed that the expected maximum Sharpe ratio of a set of independent random strategies grows with the number of configurations tried, so a backtest reported without the number of trials is uninformative. Our design is exposed to that mechanism: each run evaluates 984 candidate configurations of a feature gate and eight continuous controls against a validation block of 208 days, and eleven optimizers are compared. We therefore treat the validation objective as a search criterion rather than as evidence, and we report the untouched-holdout distribution over repeated runs instead of the best run.

The same argument motivates strong baselines. Bergstra and Bengio (2012) showed that random search is a competitive tuning procedure whenever only a few dimensions of the search space matter, which makes it the natural control for a metaheuristic comparison. On the financial side, volatility-managed exposure is a simple rule that improves risk-adjusted returns in many samples (Moreira and Muir, 2017), and buy-and-hold remains the reference that any defensive overlay has to beat after transaction costs. Section 4.5 compares the optimized policies against both.

### 2.3 Compared metaheuristics and multi-objective evaluation

In Kennedy and Eberhart (1995), particle swarm optimization was introduced through personal and social information exchange. In Karaboga and Basturk (2007), artificial bee colony optimization was formulated around employed, onlooker, and scout phases. In Holland (1975), genetic search was developed through selection, recombination, and mutation. In Yang and Deb (2009), cuckoo search combined replacement with Levy-flight exploration. In Arora and Singh (2019), butterfly optimization used fragrance-driven local and global movements. In Mirjalili and Lewis (2016), whale optimization modeled encircling, exploration, and spiral behavior. In Trojovsky and Dehghani (2022), pelican optimization represented movement toward prey followed by local refinement. In Mirjalili et al. (2014), gray wolf optimization used alpha, beta, and delta leaders. In Rao et al. (2011), teaching-learning-based optimization alternated teacher and learner phases with few algorithm-specific parameters. In Storn and Price (1997), differential evolution generated scale-adaptive perturbations from population differences. In Dorigo and Gambardella (1997), ant colony optimization accumulated distributed search experience through pheromone-like probabilities.

The comparison must be repeated because stochastic algorithms can produce favorable single runs that are not representative. In Nadimi-Shahraki et al. (2023), the authors found that WOA variants often trade improvement on one benchmark for weaker robustness on another. In Cao and Huang (2024), the authors showed that additional BOA strategies can improve exploration but increase algorithmic complexity. In Liang et al. (2024), the authors demonstrated that dynamic gain mechanisms can accelerate WOA convergence, yet application performance remains objective-dependent. In Yong et al. (2025), the authors improved POA for parameter identification and highlighted sensitivity to premature convergence. We therefore keep every optimizer non-hybrid and place the complexity in a shared, transparent financial objective system.

Hypervolume measures the dominated objective-space volume Zitzler et al., (2003). Inverted generational distance measures proximity to a pooled reference front Ishibuchi et al., (2015). Friedman (1937) ranks provide a distribution-free comparison across repeated runs. Demsar (2006) recommended this combination for the comparison of several methods over several problems and warned that an omnibus rejection is not evidence for any particular pairwise claim. Iman and Davenport (1980) proposed the less conservative *F* form of the Friedman statistic that we also report, the pairwise tests use the signed-rank statistic of (Wilcoxon, 1945), and Garcia and Herrera (2008) extended the recommendation to the full set of pairwise comparisons. Holm (1979)'s procedure controls family-wise error in post-hoc testing. Effect sizes follow the dominance statistic of Cliff (1993). In the first version of this manuscript the Holm-adjusted pairwise analysis was deferred to future work; it is carried out here, and it changes several of our original conclusions. These optimization indicators are combined with untouched predictive, calibration, and financial metrics so that no single statistic defines success.

## 3 Materials and methods

### 3.1 Data, features, and chronological protocol

The MATLAB pipeline used daily SPY open, high, low, close, adjusted close, and volume observations together with VIX open, high, low, and close values. The exact CSV used in the experiment is publicly available from the hackingthemarkets/vix-strategy repository and is cited in the Data Availability Statement. The validated snapshot contains 1,298 trading days from 2 January 2015 to 28 February 2020. To prevent external availability from affecting reproducibility, the same snapshot is compressed inside the supplied MATLAB file and is reconstructed automatically when the online source or local cache is unavailable.

Several predictors and the rolling stress threshold require a causal warm-up window of 252 observations, so the usable sample is smaller than the raw snapshot. It consists of 1,042 observations from 31 December 2015 to 21 February 2020, and the chronological 60/20/20 split assigns 625 days to training (31 December 2015 to 25 June 2018, 153 stress days), 208 days to validation (26 June 2018 to 24 April 2019, 69 stress days) and 209 days to the untouched test block (25 April 2019 to 21 February 2020, 48 stress days). These counts are stated explicitly because the effective validation sample, and not the raw number of trading days, limits how much adaptive model selection the design can support: 208 days and 69 positive events are used to select a feature subset out of 28 candidates and to tune eight continuous controls.

The predictor library contained one-day SPY returns; 5-, 10-, 20-, and 60-day momentum; rolling volatility; rolling drawdown; intraday range; volume change and volume volatility; VIX level, changes, and rolling z-scores; downside deviation; return-VIX correlation; distance from the 20-day moving average; and Amihud illiquidity. Every predictor was causal and backward-looking.

The future-stress response was defined as


$$\begin{array}{l} y_{t}=I\left[\underset{1\leq h\leq H}{\max}\text{VI}{\text{X}}_{t+h}\geq Q_{q,t}^{\left(W\right)}\left(\text{VIX}\right)\right], \end{array}$$ (1)

where *H* = 5 trading days, *W* = 252 historical observations, and *q* = 0.80. The threshold $Q_{q,t}^{\left(W\right)}$ used information available no later than date *t*. The model therefore predicts whether the market will enter an unusually high-volatility state during the next trading week rather than explaining an event after it occurs.

Two properties of Equation 1 matter for the interpretation of the results. The construction is causal, since the threshold uses past VIX values only and the label uses future values of the VIX exclusively through the outcome. The label is nevertheless defined on the same variable that supplies six of the 28 candidate predictors, so a model can exploit the persistence of the VIX itself. This is not leakage, but it makes a univariate rule based on the current VIX level the natural reference. On the untouched holdout that rule reaches an area under the curve of 0.772, and every multivariate result below is compared against it.

Observations were divided chronologically into 60% training, 20% validation, and 20% untouched test periods. Model coefficients were estimated on training data. Indicator selection, probability threshold, calibration temperature, allocation controls, and Pareto compromise selection used validation data only. The test period was accessed once after all choices had been frozen. No quantity computed on the test period was used to select an algorithm, a run, a compromise solution or a hyper-parameter. The run displayed as representative in Table 1 is chosen by the validation-space rule of the supplementary code, and Panel C of the same table reports the complete distribution over runs so that no conclusion depends on that choice.

Table 1Untouched-holdout evidence.

**Table:** 

Method	Acc.	Bal. acc.	Sens.	Spec.	Prec.	F1	AUC	Brier	ECE
Panel A. Predictive performance and probability calibration of the representative run									
PSO	0.756	0.717	0.646	0.789	0.477	0.549	0.790	0.207	0.173
ABC	0.732	0.687	0.604	0.770	0.439	0.509	0.760	0.203	0.184
GA	0.732	0.702	0.646	0.758	0.443	0.525	0.779	0.182	0.137
CS	0.742	0.715	**0.667**	0.764	0.457	0.542	**0.791**	0.184	0.154
BOA	**0.799**	0.709	0.542	**0.876**	**0.565**	0.553	0.766	0.188	0.172
WOA	0.751	0.685	0.562	0.807	0.466	0.509	0.740	0.201	0.177
POA	0.766	**0.724**	0.646	0.801	0.492	**0.559**	0.778	0.180	0.146
GWO	0.751	0.722	**0.667**	0.776	0.471	0.552	0.770	0.203	0.180
TLBO	0.746	0.689	0.583	0.795	0.459	0.514	0.765	0.198	0.166
DE	0.722	0.681	0.604	0.758	0.426	0.500	0.768	0.203	0.173
ACO	0.737	0.690	0.604	0.776	0.446	0.513	0.770	0.207	0.173
RS^†^	0.732	0.687	0.604	0.770	0.439	0.509	0.773	**0.173**	**0.120**

**Table:** 

Method	Features	Return (%)	Sharpe	Max DD (%)	Ann. vol. (%)	Turnover	Mean exposure
Panel B. Defensive-allocation performance of the same solutions							
PSO	8	7.15	1.270	−5.83	6.74	18.54	0.679
ABC	11	7.62	1.214	−5.91	7.53	26.72	0.735
GA	5	3.93	0.666	−5.00	7.39	13.90	0.682
CS	9	**12.02**	**1.754**	**−3.83**	7.99	33.30	0.723
BOA	4	8.28	1.421	−5.44	6.92	17.88	0.679
WOA	4	−1.39	−247	−5.13	6.09	15.00	0.517
POA	9	11.11	1.728	−4.62	7.52	22.12	0.733
GWO	9	6.34	1.086	−5.02	7.06	18.85	0.687
TLBO	8	5.47	0.801	−4.93	8.46	21.17	0.738
DE	10	8.80	1.400	−6.33	7.46	25.97	0.723
ACO	9	5.32	0.872	−5.94	7.49	18.22	0.732
RS^†^	12	6.07	0.918	−4.71	8.09	21.01	0.769

**Table:** 

Method	Bal. acc.	95% CI	Sensitivity	AUC	F1	Return (%)	95% CI	Sharpe	Features
Panel C. Distribution of the holdout outcome over all 30 runs									
PSO	0.677±.036	[0.664, 0.689]	0.540±.093	0.757±.024	0.496±.050	2.85 ± 4.47	[1.28, 4.39]	0.518±.697	6.3
ABC	0.671±.028	[0.661, 0.681]	0.535±.084	0.749±.020	0.488±.038	2.58 ± 3.58	[1.30, 3.79]	0.428±.556	6.0
GA	0.678±.025	[0.669, 0.686]	0.564±.101	0.756±.023	0.496±.032	3.35 ± 2.41	[2.48, 4.16]	0.517±.366	7.7
CS	0.693±.026	[0.684, 0.702]	0.638±.112	0.763±.019	0.514±.032	5.67 ± 2.53	[4.80, 6.60]	0.933±.458	9.4
BOA	0.697±.033	[0.685, 0.708]	0.694±.101	0.756±.042	0.514±.037	4.34 ± 3.49	[3.11, 5.56]	0.748±.565	4.1
WOA	0.688±.036	[0.675, 0.700]	0.665±.108	0.748±.037	0.505±.044	3.27 ± 3.76	[1.93, 4.57]	0.590±.709	4.5
POA	0.690±.023	[0.682, 0.698]	0.603±.078	0.756±.022	0.512±.029	2.57 ± 4.06	[1.17, 3.99]	0.452±.665	4.4
GWO	0.671±.029	[0.661, 0.682]	0.529±.089	0.751±.020	0.488±.040	1.76 ± 3.97	[0.38, 3.17]	0.309±.668	4.4
TLBO	0.674±.034	[0.663, 0.686]	0.557±.103	0.746±.027	0.491±.045	1.68 ± 3.84	[0.37, 3.04]	0.333±.668	4.4
DE	0.661±.034	[0.649, 0.673]	0.509±.097	0.745±.024	0.473±.049	2.26 ± 4.38	[0.67, 3.80]	0.378±.657	6.3
ACO	0.662±.027	[0.653, 0.672]	0.508±.074	0.747±.023	0.477±.037	2.69 ± 3.36	[1.51, 3.93]	0.448±.529	5.2
RS^†^	0.681±.030	[0.670, 0.691]	0.588±.099	0.754±.027	0.500±.040	4.53 ± 2.47	[3.70, 5.44]	0.694±.375	11.4

Panels A and B describe the validation-selected compromise of the representative run of every method; Panel C describes the same quantities over all 30 independent runs.

The representative run is selected by the validation-space rule of the supplementary code (0.75 normalized scalar loss and 0.25 normalized hypervolume); the holdout was never used for that choice. Confidence intervals are percentile bootstrap intervals with 10 000 resamples. Friedman tests over the 30 paired runs (BA: $\chi_{F}^{2}=43.11$, *p* = 4.76 × 10^−6^, Se: $\chi_{F}^{2}=89.59$, *p* = 6.45 × 10^−15^, AUC: $\chi_{F}^{2}=23.38$, *p* =.0094, F1: $\chi_{F}^{2}=35.80$, *p* = 9.11 × 10^−5^, Return: $\chi_{F}^{2}=27.38$, *p* =.0023, Sharpe: $\chi_{F}^{2}=25.37$, *p* =.0047). ^†^RS is the uniform random-search control. The spread between the columns of Panels A and B is of the same order as the run-to-run dispersion reported in Panel C.

Bold values indicate the best value in each directly comparable performance column (ties included; lower Brier, ECE, and |Max DD| are better).

Figure 1a shows a market environment with persistent upward price movement interrupted by distinct volatility clusters, abrupt VIX spikes, volume expansion, and widening intraday ranges. This co-movement supports a multivariate representation because price, implied volatility, participation, and liquidity carry complementary information. Figure 1b shows that the forward labels concentrate around persistent volatility expansion rather than isolated daily losses, which a one-day return threshold would miss. Del Fava et al. (2024) found that predictability changes with the forecast horizon, and Campisi et al. (2024) showed that volatility indices contain predictive information for equity-market direction; the five-day label combines both insights with a rolling historical threshold that adapts to the risk level known at each prediction date.

**Figure 1:**
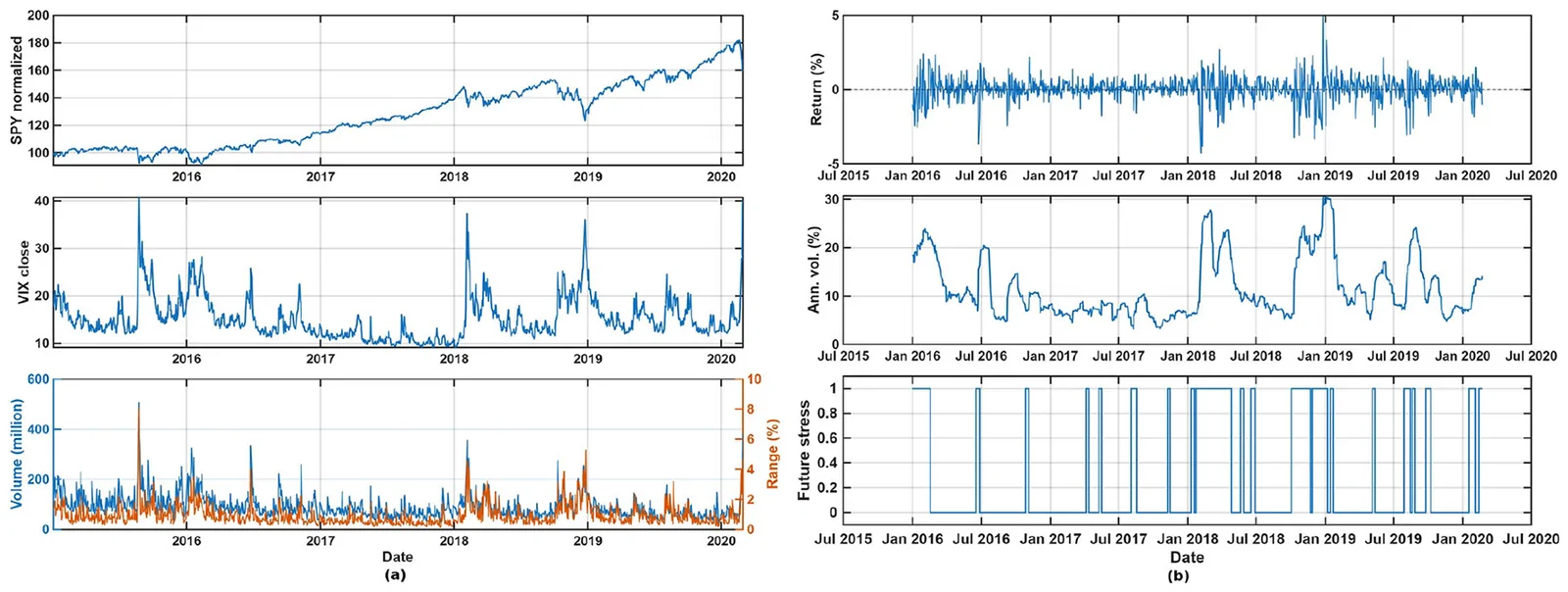
Market environment and construction of the response. **(a)** Chronological SPY and VIX overview: the normalized equity path, VIX level, trading volume and intraday range display volatility clustering, participation shocks and abrupt transitions. **(b)** Construction of the five-day forward response: daily returns and backward-looking annualized volatility are shown with the binary label of Equation 1; only the response uses the subsequent five trading days.

### 3.2 Mixed model–policy decision vector

Each candidate solution contained a continuous gate for every candidate feature and eight normalized continuous controls:


$$\begin{array}{c} \text{x}=\left[z_{1},\ldots ,z_{M},u_{\lambda },u_{\tau },u_{T},u_{e},u_{\gamma },u_{\sigma },u_{\kappa },u_{d}\right]\in {\left[0,1\right]}^{M+8}. \end{array}$$ (2)

A feature was active when *z*_*j*_>0.5, subject to 4–12 selected predictors. The normalized genes were decoded as


$$\begin{array}{c} \lambda =10^{-5+7u_{\lambda }},\;\tau =0.10+0.75u_{\tau },\;T=0.50+2.50u_{T}, \end{array}$$ (3)

$$\begin{array}{c} e_{\min}=0.60u_{e},\;\gamma =0.50+3.50u_{\gamma },\sigma^{*}=0.08+0.22u_{\sigma }, \end{array}$$ (4)

$$\begin{array}{c} \kappa =0.95u_{\kappa },\;d_{\text{cut}}\;=0.03+0.17u_{d}. \end{array}$$ (5)

Here, λ is ridge regularization, τ is the classification threshold, *T* is the temperature-scaling coefficient, *e*_min_ is minimum market exposure, γ maps stress probability to de-risking intensity, σ^*^ is the annualized volatility target, κ smooths exposure, and *d*_cut_ is a drawdown-based de-risking trigger. Equations 2–11 collectively define the mixed model-policy decision vector, its decoded controls, and the first six minimized objectives. The upper bound of *e*_min_ and the weight of the Brier term in Equation 12 are corrected here to the values used by the implementation throughout; the first version printed 0.80 in both places. All numerical results of both versions were produced with the values printed above.

### 3.3 Seven-objective formulation

A class-weighted ridge-logistic model was fitted for every repaired decision vector. Let balanced accuracy be BA, sensitivity be Se, F1-score be *F*_1_, area under the ROC curve be *A*, Brier score be *B*, expected calibration error be *E*, the number of selected features be *m*, and the total candidate count be *M*. The seven minimized objectives were


$$\begin{array}{c} f_{1}=1-\left(0.45\text{BA}+0.30A+0.25F_{1}\right), \end{array}$$ (6)

$$\begin{array}{c} f_{2}=1-\text{Se}, \end{array}$$ (7)

$$\begin{array}{c} f_{3}=\min\left\{1,0.60B+0.40E\right\}, \end{array}$$ (8)

$$\begin{array}{c} f_{4}=m/M, \end{array}$$ (9)

$$\begin{array}{c} f_{5}=\min\left\{1,\left(1-\text{B}{\text{A}}_{\text{worst}}\right)+0.25s_{\text{BA}}\right\}, \end{array}$$ (10)

$$\begin{array}{c} f_{6}=\min\left\{1,|\text{MDD}|/0.25\right\}, \end{array}$$ (11)

$$\begin{array}{c} f_{7}=0.50\left(1-\tilde{S}\right)+0.30\left(1-\tilde{R}\right)+0.20\min\left\{1,\text{TO}/1.5\right\}, \end{array}$$ (12)

where BA_worst_ and *s*_BA_ quantify the weakest and dispersed validation-block performance, MDD is maximum drawdown, $\tilde{S}$ and $\tilde{R}$ are normalized Sharpe and return benefits, and TO is turnover. The scalar validation loss used only for representative compromise selection was


$$\begin{array}{c} L\left(\text{x}\right)=0.22f_{1}+0.18f_{2}+0.12f_{3}+0.08f_{4}+0.15f_{5}+0.12f_{6}+0.13f_{7}, \end{array}$$ (13)

augmented by quadratic penalties that discourage solutions with a validation sensitivity below 0.55, a predictive loss above 0.40 or a drawdown loss above 0.60. The Pareto archive remained seven-dimensional; Equation 13 did not replace dominance-based search.

### 3.4 Defensive allocation policy

The calibrated stress probability *p*_*t*_ was transformed into a causal exposure *e*_*t*_∈[*e*_min_, 1]. Higher stress probability reduced exposure according to γ, while the volatility target and drawdown trigger imposed additional risk controls. The smoothed exposure was applied to the next-period SPY return, and each absolute exposure change incurred a transaction cost of 5 basis points. The strategy could therefore not act on a return before its corresponding probability was available.

### 3.5 Compared optimizers and workflow

The comparison included PSO, ABC, GA, CS, BOA, WOA, POA, GWO, TLBO, DE, and ACO. All methods received the same population, maximum iterations, objective function, feature limits, archive capacity, and random-seed policy. Every method used a population of 24 candidates and 40 iterations, so each run performed 984 objective evaluations, and the experiment was repeated over 30 independent runs per method, three times the number reported in the first submission. The non-dominated archive removed duplicates, retained objective-space diversity, and was used to calculate hypervolume, IGD, and spacing.

A uniform random-search control was added in this revision. It draws the same number of candidate solutions from the uniform distribution over the decision space, repairs them with the same feature-cardinality rules, and stores them in the same non-dominated archive under an identical evaluation budget and the same 30 runs. Any advantage a metaheuristic shows over this control is attributable to its search mechanism; any advantage that both share over a fixed model is attributable to the amount of tuning rather than to the optimizer.

Figure 2 shows the end-to-end decision pipeline. The same candidate solution controls the feature subset, classifier regularization, probability threshold, calibration temperature, minimum exposure, risk exponent, volatility target, smoothing coefficient, and drawdown trigger. The seven objectives are evaluated on validation data and stored in a non-dominated archive. Only after the search is complete is one compromise evaluated on the untouched test set, which prevents the backtest from influencing model development.

**Figure 2:**
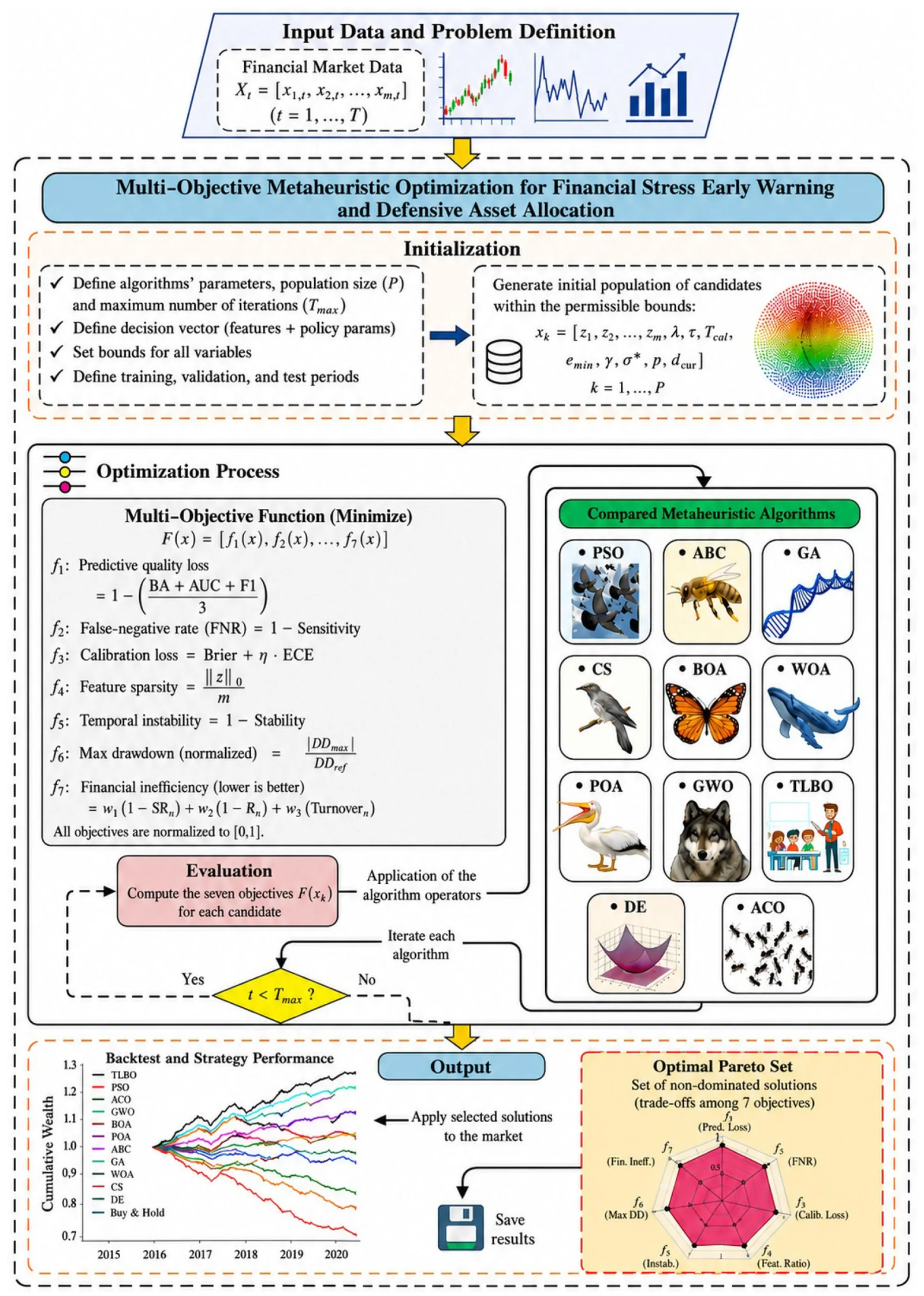
Leakage-controlled multi-objective framework linking volatility-regime forecasting, probability calibration, Pareto search, validation-based compromise selection, and causal defensive allocation.

### 3.6 Reference strategies

Nine reference strategies are evaluated on exactly the same untouched holdout window, with the same 5 basis-point cost per unit of absolute exposure change and the same causal timing convention: buy-and-hold SPY; two constant-exposure portfolios at 60% and 80%; two volatility-targeting rules that scale exposure toward an annualized target of 10% and 15% using a trailing 20-day volatility estimate and no predictive model; two VIX-threshold rules that cut exposure to zero or to 30% whenever the VIX exceeds the same rolling 80th percentile that defines the label; a ridge-logistic model estimated on every candidate predictor with a penalty selected on the validation block alone, a fixed 0.50 decision threshold and binary de-risking; and the same ridge-logistic probabilities inside a hand-specified defensive policy with *e*_min_ = 0.20, γ = 2, σ^*^ = 15%, κ = 0.50 and *d*_cut_ = 0.10. None of the nine involves any search over the decision space, so they measure how much of the reported economic performance requires optimization at all.

### 3.7 Rolling-origin evaluation

A single chronological holdout answers one question about one market window. The whole pipeline is therefore repeated at five successive origins. For each origin the development block consists of every observation before the origin, split 75/25 into training and validation, and the following 104 trading days form an untouched test block. The five test blocks are consecutive and non-overlapping, cover 26 January 2018 to 21 February 2020, and have stress rates between 0.219 and 0.705, which spans a calm expansion, the February 2018 volatility shock and the late-2018 correction. Ten independent runs of every optimizer and of the random-search control are performed at each origin, giving 50 (origin, run) blocks per method.

### 3.8 Alternative stress definitions and predictor ablation

Five additional configurations were optimized from scratch under the identical protocol. In the first, the label of Equation 1 is retained but the six VIX-derived predictors are removed, leaving 22 price, volume, drawdown and liquidity variables. In the remaining four, the predictor library is unchanged and the driver of Equation 1 is replaced by the five-day forward maximum of, respectively, a 5-day realized volatility, the depth of a 10-day SPY drawdown, a five-day average Amihud illiquidity, and an equally weighted composite of standardized implied volatility, realized volatility, drawdown depth and illiquidity. Every variant keeps the rolling 80th-percentile threshold, the five-day horizon and the causal warm-up rule. Eleven optimizers and ten runs were used for each variant, so each reported value pools 110 untouched-holdout evaluations.

### 3.9 Reproducibility and supplementary implementation

The complete self-contained MATLAB program is supplied as Supplementary File 1. It downloads the public SPY-VIX CSV when internet access is available, validates every file before use, reconstructs an embedded snapshot when necessary, creates all engineered predictors, runs the eleven optimizers, performs repeated-run statistical analysis, exports every table, and saves editable MATLAB figures. Supplementary File 2 adds the random-search control, the 30-run configuration, the Holm-adjusted post-hoc analysis with effect sizes and bootstrap intervals, the nine reference strategies, the rolling-origin driver and the five alternative stress definitions, so that every number in this paper can be regenerated by a single script.

### 3.10 Evaluation and statistical analysis

Holdout classification was assessed using accuracy, balanced accuracy, sensitivity, specificity, precision, F1-score, and ROC-AUC. Probability quality was assessed through the Brier score and 10-bin expected calibration error. Financial evaluation used total return, annualized Sharpe ratio, maximum drawdown, annualized volatility, turnover, and mean exposure. Optimization quality was measured by hypervolume, IGD, nearest-neighbor spacing, archive size, runtime, and scalar validation loss.

Thirty independent runs were performed for every method. Omnibus differences across the eleven metaheuristics were tested with the Friedman statistic and with its Iman–Davenport *F* transformation at α = 0.05. Whenever the omnibus test rejected equality, all 55 pairwise comparisons were carried out with the Wilcoxon signed-rank test on the paired runs, and the resulting *p*-values were adjusted with Holm's step-down procedure within each indicator family. Two effect sizes accompany every comparison: the matched-pairs rank-biserial correlation and Cliff's δ. Percentile bootstrap confidence intervals with 10,000 resamples are reported for the paired mean differences and for every mean reported over runs. The comparison of each method against the random-search control is treated as a separate family of eleven tests and corrected accordingly.

## 4 Results

Every table and figure that carries a number was regenerated for this revision from the 30-run experiment described in Section 3, so the values differ from the first submission, which rested on ten runs. The material is now organized into six multi-panel tables and nine figures.

### 4.1 Holdout performance and its dispersion across runs

Panels A and B of Table 1 report the untouched holdout outcome of the validation-selected compromise of the representative run of each method, which is the protocol used in the first submission. Taken on their own, those two panels support the conclusion drawn previously: POA reaches the highest balanced accuracy (0.724), CS the largest holdout return (12.02%) and the best Sharpe ratio (1.754), and the distance between the best and the worst method looks substantial. Panel C shows why that reading is unsafe. Computed for all 30 runs of every method, the mean holdout balanced accuracy of the eleven metaheuristics lies between 0.662 (ACO) and 0.697 (BOA), while the run-to-run standard deviation of a single method is between 0.023 and 0.036. The between-method spread and the within-method dispersion are of the same order. The best single run of almost every method reaches a balanced accuracy above 0.73 and a holdout return between 8% and 12%, while its weakest run falls below 0.62 and loses money. Selecting the favorable run after the fact produces large numbers that carry no information about the next market window.

The Friedman test over the 30 paired runs rejects equality of the eleven methods for holdout balanced accuracy ($\chi_{F}^{2}=43.11$, *p* = 4.8 × 10^−6^) and for sensitivity ($\chi_{F}^{2}=89.59$, *p* = 6.5 × 10^−15^), which reflects a difference in operating point rather than in ranking quality: BOA, CS and WOA settle on lower decision thresholds and recover more stress days, whereas ACO, DE and GWO settle on more conservative thresholds. The omnibus test is much weaker for the ranking itself ($\chi_{F}^{2}=23.38$, *p* = 0.0094 for the area under the curve), and the pairwise picture is thin. After Holm correction only seven of the 55 balanced-accuracy comparisons survive, the largest being BOA over ACO (+0.034, 95% CI [0.022, 0.047], $p_{\text{Holm}}=1.1\times 10^{-3}$, Cliff's δ = 0.66); four of the 55 Sharpe comparisons survive and all involve CS; none of the 55 comparisons of the area under the curve survives. Panels B and C of Table 2 and Figure 3 give the complete picture.

Table 2Repeated-run optimisation quality over 30 independent runs, omnibus tests and Holm-corrected post-hoc analysis.

**Table:** 

Method	HV mean±SD	IGD mean±SD	Spacing mean±SD	Loss mean±SD	Runtime (s)	Archive	Opt. rank
Panel A. Optimisation indicators in the validation objective space							
GWO	0.252±.024	**.246±.033**	0.0830±.0112	0.1842±.0070	4.62	120.0	**3.929**
PSO	0.248±.030	0.269±.031	0.0741±.0112	0.1880±.0142	4.76	120.0	4.213
ABC	0.255±.019	0.290±.022	0.0757±.0091	0.1903±.0067	4.33	116.7	4.558
ACO	**.258±.025**	0.308±.022	0.0798±.0118	**.1839±.0076**	4.67	118.8	4.817
DE	0.253±.025	0.300±.023	0.0777±.0107	0.1908±.0056	4.60	117.2	5.117
TLBO	0.244±.030	0.287±.020	0.0810±.0111	0.1900±.0104	4.45	119.8	5.138
POA	0.244±.024	0.308±.038	0.0711±.0124	0.2014±.0070	4.77	119.6	5.425
BOA	0.195±.021	0.313±.031	**.0673±.0083**	0.2304±.0083	4.40	117.8	7.067
WOA	0.186±.024	0.309±.032	0.0896±.0191	0.2255±.0151	4.56	118.4	8.054
CS	0.176±.020	0.362±.028	0.0685±.0085	0.2382±.0162	4.57	119.4	8.258
GA	0.152±.025	0.435±.064	0.0842±.0207	0.2300±.0196	4.46	95.4	9.425
RS^†^	0.175±.011	0.385±.031	0.0719±.0078	0.2425±.0102	4.64	120.0	–

**Table:** 

Indicator	$\chi_{F}^{2}$	*p* (Friedman)	*F*_*ID*_	*p* (Iman–Davenport)	Holm-significant pairs
Panel B. Omnibus tests across the eleven metaheuristics and surviving pairwise comparisons					
Hypervolume (validation)	206.53	7.00 × 10^−39^	64.08	1.65 × 10^−67^	31/55
IGD (validation)	172.46	8.58 × 10^−32^	39.21	3.01 × 10^−48^	33/55
Spacing (validation)	63.76	6.98 × 10^−10^	7.83	4.00 × 10^−11^	13/55
Scalar validation loss (validation)	226.74	4.14 × 10^−43^	89.75	1.09 × 10^−82^	39/55
Balanced accuracy (holdout)	43.11	4.76 × 10^−6^	4.87	1.70 × 10^−6^	7/55
ROC area (holdout)	23.38	.0094	2.45	.0080	0/55
Sharpe ratio (holdout)	25.37	.0047	2.68	.0038	4/55

**Table:** 

	Pairwise wins/losses				vs. random search (Cliff's δ)			
Method	HV	Loss	Holdout BA	Holdout Sharpe	HV	Loss	Holdout BA	Holdout Sharpe
Panel C. Significant wins/losses out of ten pairwise tests, and comparison with the random-search control								
PSO	4/0	5/0	0/0	0/0	better (1.00)	better (−1.00)	ns (−0.04)	ns (−0.19)
ABC	4/0	5/1	0/0	0/1	better (1.00)	better (−1.00)	ns (−0.19)	ns (−0.29)
GA	0/10	0/7	0/0	0/0	worse (−0.52)	better (−0.40)	ns (−0.09)	ns (−0.18)
CS	1/7	0/8	2/0	4/0	ns (−0.02)	ns (−0.16)	ns (0.19)	ns (0.34)
BOA	1/7	0/7	3/0	0/0	better (0.63)	better (−0.64)	ns (0.32)	ns (0.11)
WOA	1/7	1/7	0/0	0/0	ns (0.30)	better (−0.64)	ns (0.19)	ns (−0.08)
POA	4/0	4/6	2/0	0/0	better (0.99)	better (−1.00)	ns (0.17)	ns (−0.26)
GWO	4/0	8/0	0/1	0/0	better (1.00)	better (−1.00)	ns (−0.20)	ns (−0.43)
TLBO	4/0	5/1	0/0	0/1	better (0.99)	better (−1.00)	ns (−0.10)	ns (−0.38)
DE	4/0	5/2	0/3	0/1	better (1.00)	better (−1.00)	ns (−0.34)	ns (−0.34)
ACO	4/0	6/0	0/3	0/1	better (1.00)	better (−1.00)	ns (−0.41)	ns (−0.34)

Hypervolume is maximized; IGD, spacing, loss, runtime and ranks are minimized. Degrees of freedom are 10 for every Friedman test and (10, 290) for every Iman–Davenport test. Pairwise tests are Wilcoxon signed-rank tests on the paired runs with Holm correction inside each indicator family; the random-search comparison is corrected as a separate family of eleven tests. The random-search control is excluded from the ranks and from the omnibus tests. Runtimes were measured on two shared virtual cores. Bold values indicate the best optimization metric (HV maximized; IGD, spacing, scalar loss, and optimization rank minimized). ^†^RS denotes the uniform random-search control.

**Figure 3:**
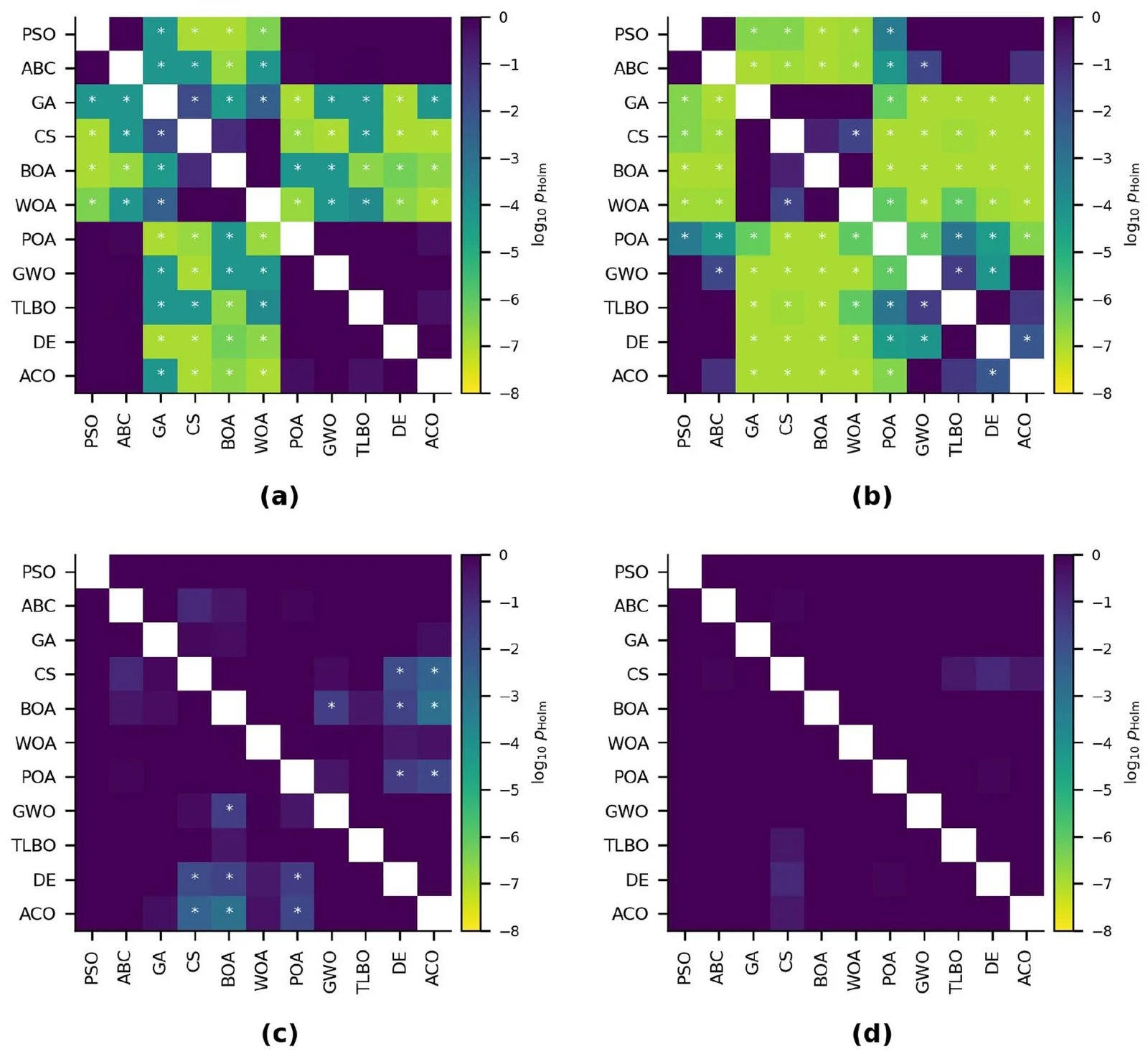
Holm-adjusted *p*-values of all 55 pairwise Wilcoxon signed-rank comparisons over the 30 paired runs: **(a)** hypervolume and **(b)** scalar validation loss in the validation space, **(c)** balanced accuracy and **(d)** ROC area on the untouched holdout. Asterisks mark comparisons that survive Holm correction at α = 0.05. The contrast between the upper and the lower row is the main statistical result of the study.

### 4.2 Validation trade-offs and optimized decision parameters

Panel A of Table 3 confirms that the seven objectives conflict. Solutions with a similar predictive loss differ substantially in missed-event risk, calibration, sparsity, stability, drawdown and financial inefficiency, and no method occupies the best position on more than a few coordinates at once. Panel B shows how much freedom the search has: the decision threshold ranges from 0.294 to 0.663, the minimum exposure from 0 to 0.131, the volatility target from 8% to 30% and the smoothing coefficient from 0 to 0.508, all at a comparable scalar validation loss. Eight continuous controls and a 28-dimensional feature gate reach a similar validation profile along many different paths, so validation performance stops being a reliable guide to out-of-sample behavior. Panel C reports the correlation structure of the objectives: predictive loss is associated with calibration loss (*r* = 0.598) and with financial inefficiency (*r* = 0.632), calibration loss with financial inefficiency (*r* = 0.589), and maximum-drawdown loss with financial inefficiency (*r* = 0.457) and with the false-negative rate (*r* = 0.436). The relationships are substantial but far from perfect, which is what justifies keeping the objectives separate. The false-negative rate has only a weak relationship with aggregate predictive loss (*r* = −0.089), so a model can achieve a favorable combined prediction score while missing important events, and temporal instability is negatively correlated with every other objective.

Table 3Validation objective space: selected trade-off vectors, optimized model and policy controls, and the dependence structure of the seven objectives.

**Table:** 

Method	Pred. loss	FNR	Calib. loss	Feature ratio	Temporal instab.	DD loss	Fin. ineff.	Scalar loss
Panel A. Seven-objective vectors of the representative solutions and weighted compromise loss								
PSO	0.0641	0.0145	0.1162	0.2857	0.3272	0.1243	0.3046	0.1571
ABC	0.1051	0.0290	0.1223	0.3929	0.3267	0.1266	0.2999	0.1776
GA	0.1119	0.0145	0.1101	0.1786	0.5630	0.0908	0.4212	0.2048
CS	0.1026	0.0435	0.1259	0.3214	0.4784	0.1447	0.3490	0.2057
BOA	0.1246	0.1739	0.1283	0.1429	0.3038	0.2112	0.4456	0.2144
WOA	0.1013	0.0145	0.0961	0.1429	0.5648	0.0821	0.4256	0.1977
POA	0.0966	0.0290	0.1268	0.3214	0.4917	0.0985	0.2644	0.1873
GWO	0.0928	0.0290	0.1219	0.3214	0.3290	0.0963	0.2684	0.1618
TLBO	0.0955	0.0580	0.1174	0.2857	0.3308	0.1252	0.2502	0.1656
DE	0.0959	0.0145	0.1191	0.3571	0.3216	0.1073	0.3479	0.1729
ACO	0.0857	0.0435	0.1191	0.3214	0.2597	0.1194	0.2551	0.1531
RS^†^	0.1071	0.0145	0.1116	0.4286	0.5645	0.1037	0.3842	0.2209

**Table:** 

Method	*m*	λ	τ	*T*	*e*_min_	γ	σ^*^	κ	*d*_cut_
Panel B. Optimized model, calibration and defensive-policy controls									
PSO	8	0.00846	0.588	0.500	0.000	3.992	0.193	0.355	0.049
ABC	11	10^−4.04^	0.463	0.500	0.039	2.823	0.298	0.083	0.121
GA	5	0.0755	0.371	0.597	0.015	3.971	0.123	0.508	0.120
CS	9	10^−5.00^	0.493	0.500	0.000	3.297	0.165	0.010	0.130
BOA	4	0.00243	0.663	0.500	0.131	3.082	0.090	0.330	0.076
WOA	4	0.00729	0.294	0.500	0.000	3.218	0.080	0.000	0.030
POA	9	10^−3.26^	0.465	0.545	0.000	4.000	0.167	0.130	0.200
GWO	9	10^−3.34^	0.487	0.548	0.002	3.869	0.133	0.246	0.097
TLBO	8	0.155	0.542	0.565	0.024	4.000	0.150	0.073	0.109
DE	10	0.025	0.487	0.500	0.039	4.000	0.293	0.000	0.191
ACO	9	10^−4.32^	0.548	0.512	0.013	3.740	0.290	0.452	0.153
RS^†^	12	0.00772	0.373	0.670	0.069	3.494	0.197	0.130	0.127

**Table:** 

Objective	Pred. loss	FNR	Calib. loss	Feature ratio	Temporal instab.	DD loss	Fin. ineff.
Panel C. Pearson correlations across every archived non-dominated solution							
Predictive loss	1.000	−0.089	0.598	0.168	−0.397	0.141	0.632
False-negative rate	−0.089	1.000	−0.016	0.116	−0.121	0.436	0.054
Calibration loss	0.598	−0.016	1.000	0.318	−0.268	0.106	0.589
Feature ratio	0.168	0.116	0.318	1.000	−0.171	0.264	0.257
Temporal instability	−0.397	−0.121	−0.268	−0.171	1.000	−0.139	−0.251
Maximum-drawdown loss	0.141	0.436	0.106	0.264	−0.139	1.000	0.457
Financial inefficiency	0.632	0.054	0.589	0.257	−0.251	0.457	1.000

m: selected features; λ: ridge penalty; τ: classification threshold; *T*: calibration temperature; *e*_min_: minimum exposure; γ: risk exponent; σ^*^: annualized volatility target; κ: exposure smoothing; *d*_cut_: drawdown trigger. The scalar loss of Panel A excludes the safety penalties of Equation 13. Panel C pools every non-dominated solution produced by the eleven metaheuristics in the 30-run experiment. ^†^RS denotes the uniform random-search control.

Figures 4a, b show dense, non-identical trade-off clouds rather than one dominant frontier. Sutiene et al. (2024) concluded that portfolio-oriented AI must account for several interacting decision stages, and the objective-space evidence supports that conclusion. Abdallah et al. (2025) reported that optimizer choice changes the coverage of finance-related efficient sets; the same effect is visible here even though all methods use identical data, objectives, bounds, archive capacity and evaluation budget. Figures 4c–f further summarize repeated-run hypervolume, IGD, mean hypervolume evolution, and archive-size evolution across the compared methods.

**Figure 4:**
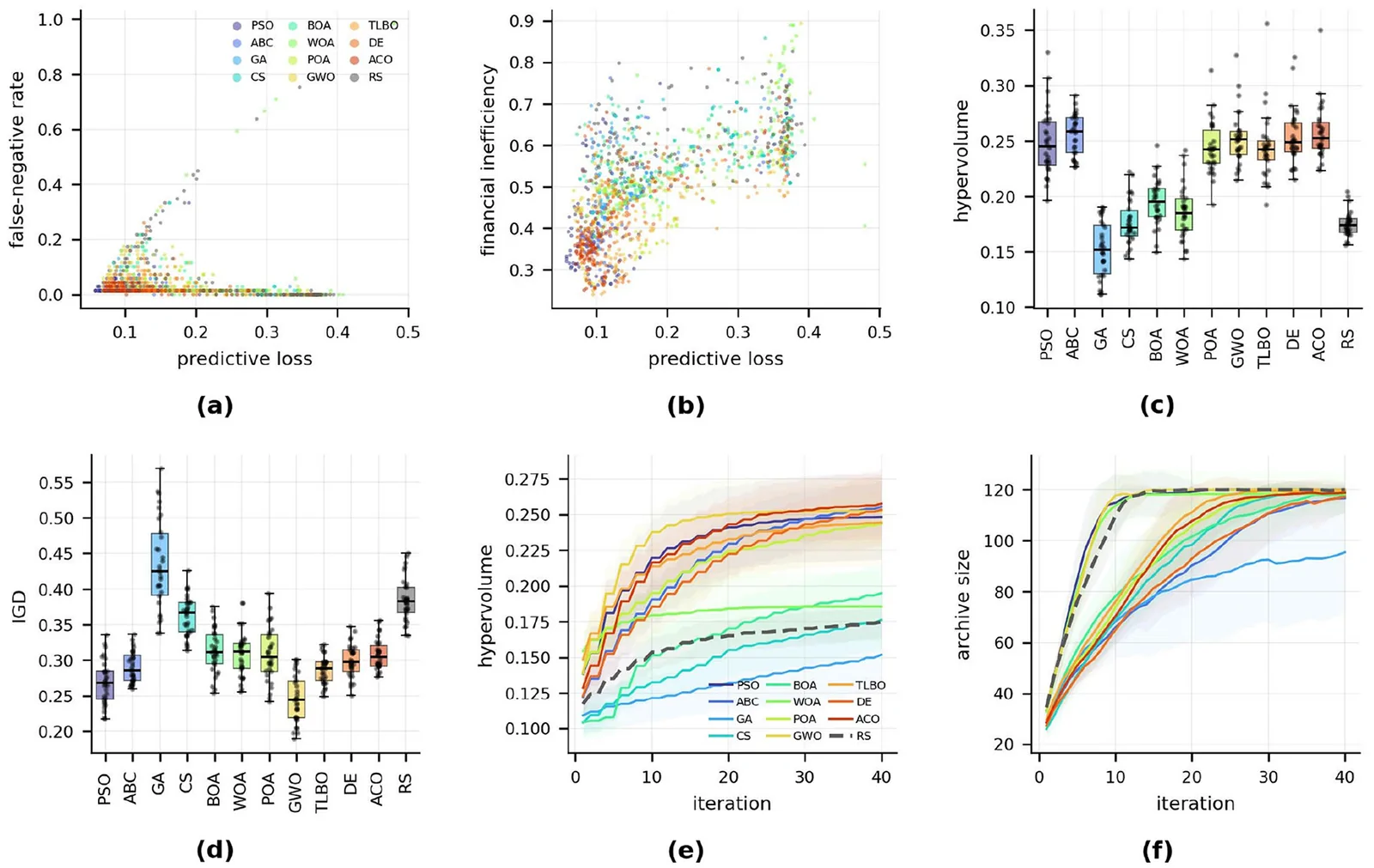
Validation objective space and search behavior. **(a)** Predictive loss against false-negative rate and **(b)** predictive loss against financial inefficiency for the archives of the representative runs. **(c)** Hypervolume and **(d)** inverted generational distance over the 30 runs of every method. **(e)** Mean hypervolume and **(f)** mean archive size against the iteration counter, with a ±1 SD band. The dashed gray curve and the gray box are the random-search control.

### 4.3 Repeated-run optimization quality

Panel A of Table 2 summarizes the 30 independent runs. In the validation objective space the algorithms behave as the metaheuristic literature predicts, and the differences are large. GWO achieves the best aggregate optimization rank (3.929) and the smallest mean IGD (0.246 ± 0.033), ACO the largest mean hypervolume (0.258 ± 0.025) and the lowest mean scalar validation loss (0.1839 ± 0.0076), and BOA the smallest nearest-neighbor spacing. GA and CS are the weakest searchers, with mean hypervolumes of 0.152 and 0.176 against 0.24–0.26 for the leading group. Friedman and Iman–Davenport tests reject equality for all four indicators, with *p*-values between 7.0 × 10^−10^ and 4.1 × 10^−43^, and 31 of the 55 hypervolume comparisons and 39 of the 55 validation-loss comparisons remain significant after Holm correction.

### 4.4 Search quality does not transfer into decision quality

The two preceding subsections describe the central result of the revised study. The eleven metaheuristics separate cleanly in the space they optimize and do not separate in the space that matters for a risk manager. Panel C of Table 2 places the two analyses side by side. Eight of the eleven methods beat the random-search control on hypervolume and ten of the eleven beat it on scalar validation loss, in both cases with Holm-adjusted *p*-values below 0.01 and a Cliff's δ of at least 0.99 for the seven strongest searchers, so essentially every one of their 30 runs beats every one of the 30 random-search runs. GA is significantly worse than random search on hypervolume, which is consistent with its narrow archives. On the untouched holdout no method differs significantly from random search on balanced accuracy, on the area under the curve or on the Sharpe ratio, and the point estimates of the differences range only from −0.020 to +0.016 balanced-accuracy points.

Figure 5c makes the relationship explicit. Plotting the mean best scalar validation loss of every method against its mean holdout balanced accuracy gives *r* = 0.71, so the methods that minimize the validation objective most effectively generalize slightly worse. GWO, ACO, TLBO and DE occupy the low-loss and low-holdout corner, while CS, BOA and the random-search control occupy the high-loss and high-holdout corner. With 208 validation days, 69 validation events and a search space containing a 28-bit feature gate and eight continuous controls, a stronger optimizer mainly buys a closer fit to one nine-month window. Figures 5a, b and 5d-f summarize aggregate optimization rank and runtime, recurrent feature-selection patterns, the selected objective vectors, and the optimized model-policy controls, respectively.

**Figure 5:**
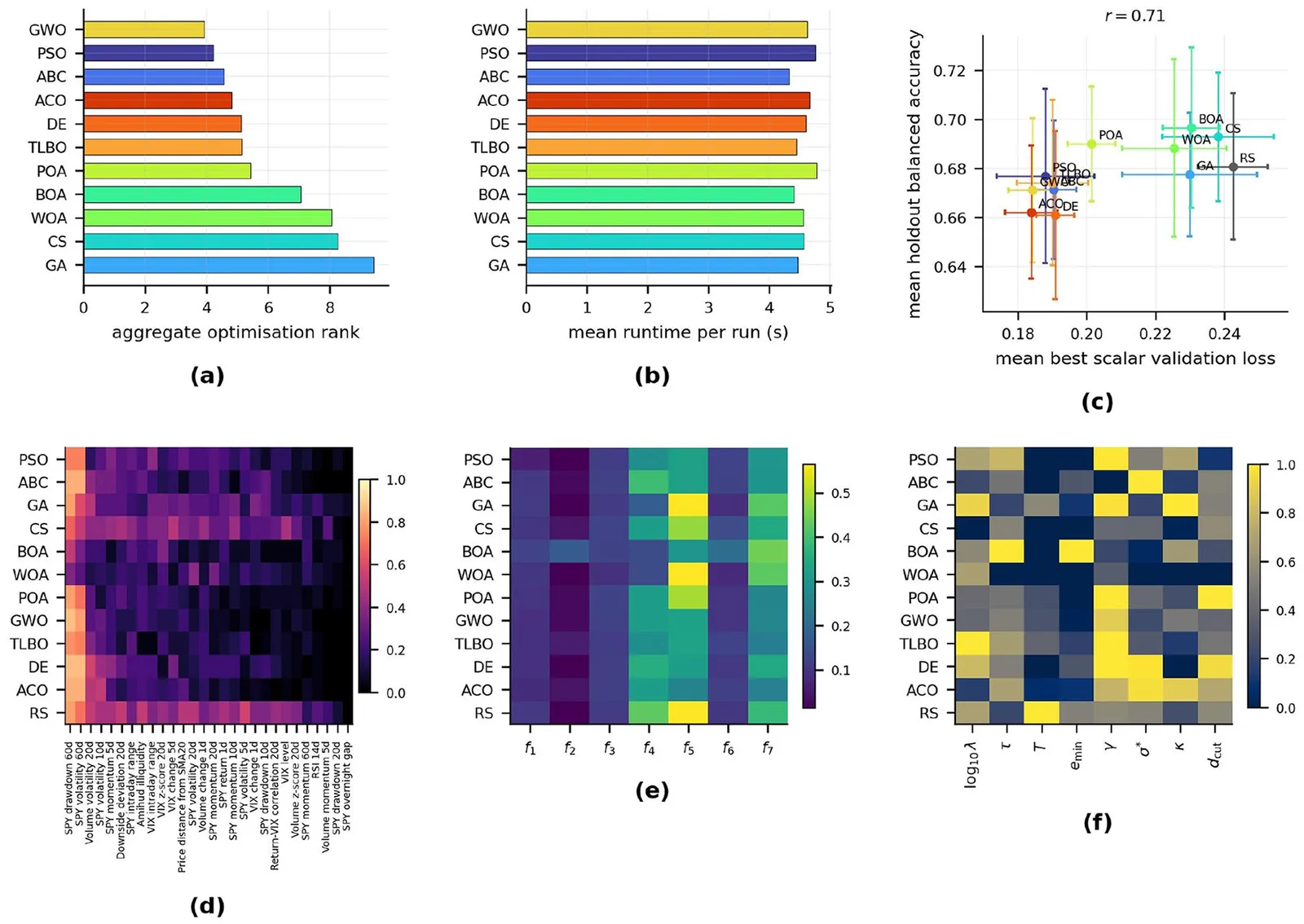
Search quality, computational cost and transfer to untouched data. **(a)** Aggregate optimisation rank and **(b)** mean runtime per run. **(c)** Mean best scalar validation loss against mean holdout balanced accuracy, with one standard deviation on both axes. **(d)** Frequency with which each candidate predictor survives feature selection across all runs. **(e)** Selected seven-objective vectors and **(f)** optimized model and policy controls of the representative solutions, min–max scaled.

Panel C of Table 4 isolates one mechanical contributor to the loss of performance between validation and holdout. The decision threshold and the calibration temperature are tuned on validation probabilities produced by a model estimated on the training block, while the coefficients are then re-estimated on training and validation together, which shifts the probability scale. Re-anchoring the threshold so that the validation alarm rate is preserved raises the mean holdout sensitivity of every method by 0.04 to 0.08 points and the mean holdout return by 0.06 to 1.22 percentage points; balanced accuracy improves by up to 1.4 points for nine of the twelve methods and falls by up to 1.2 points for the three that already used a very low threshold. The effect is systematic and worth correcting in future work, but it is an order of magnitude too small to explain the gap between validation and holdout behavior.

Table 4Supporting diagnostics: volatility regimes, recurrent predictors and the effect of the estimation protocol.

**Table:** 

Method	Low-VIX BA	Low-VIX Se	Medium-VIX BA	Medium-VIX Se	High-VIX BA	High-VIX Se
Panel A. Holdout balanced accuracy and sensitivity by VIX tercile (representative run)						
PSO	0.500	0.000	0.671	0.444	0.531	0.818
ABC	0.500	0.000	0.624	0.333	0.462	0.788
GA	0.500	0.000	0.573	0.333	0.546	0.848
CS	0.485	0.000	0.663	0.444	0.532	0.848
BOA	0.477	0.000	0.688	0.444	0.658	0.667
WOA	0.500	0.000	0.633	0.333	0.499	0.727
POA	0.500	0.000	0.641	0.333	0.532	0.848
GWO	0.500	0.000	0.577	0.222	0.522	0.909
TLBO	0.492	0.000	0.577	0.222	0.516	0.788
DE	0.500	0.000	0.599	0.333	0.475	0.788
ACO	0.500	0.000	0.633	0.333	0.462	0.788
RS^†^	0.492	0.000	0.590	0.333	0.529	0.788

**Table:** 

Rank	Predictor	Frequency	Rank	Predictor	Frequency
Panel B. Predictors most often retained across all 330 optimized models					
1	SPY drawdown 60d	73.3%	7	Downside deviation 20d	23.3%
2	SPY volatility 60d	65.8%	8	VIX intraday range	22.1%
3	Volume volatility 20d	34.2%	9	VIX change 5d	21.5%
4	SPY volatility 10d	29.7%	10	VIX z-score 20d	21.5%
5	SPY momentum 5d	25.8%	11	Amihud illiquidity	21.5%
6	SPY intraday range	23.3%	12	Volume change 1d	20.0%

**Table:** 

	A: refit on train+validation			B: no refit			C: refit, threshold re-anchored		
Method	Bal. acc.	Sens.	Return (%)	Bal. acc.	Sens.	Return (%)	Bal. acc.	Sens.	Return (%)
Panel C. Estimation protocol between validation tuning and the untouched holdout									
PSO	0.677	0.540	2.85	0.683	0.574	3.33	0.691	0.599	4.00
ABC	0.671	0.535	2.58	0.676	0.564	2.87	0.680	0.596	3.69
GA	0.678	0.564	3.35	0.685	0.641	3.34	0.687	0.648	3.79
CS	0.693	0.638	5.67	0.688	0.676	5.06	0.691	0.703	5.73
BOA	0.697	0.694	4.34	0.691	0.745	4.70	0.685	0.745	4.63
WOA	0.688	0.665	3.27	0.689	0.724	3.62	0.686	0.724	3.55
POA	0.690	0.603	2.57	0.697	0.647	2.88	0.694	0.653	3.15
GWO	0.671	0.529	1.76	0.678	0.561	2.27	0.680	0.572	2.98
TLBO	0.674	0.557	1.68	0.680	0.590	2.00	0.682	0.601	2.59
DE	0.661	0.509	2.26	0.669	0.543	1.77	0.675	0.581	3.06
ACO	0.662	0.508	2.69	0.669	0.530	2.03	0.671	0.547	2.83
RS^†^	0.681	0.588	4.53	0.679	0.637	3.93	0.681	0.658	4.85

The three holdout terciles of Panel A contain 71, 68 and 70 observations with 6, 9 and 33 stress days, so the low-VIX sensitivities rest on very few positive events. Panel B reports how often each candidate predictor survives feature selection across every optimized model of the 30-run experiment; it describes recurrence, not causal importance. Panel C reports means over the 30 runs. Protocol A is the one used throughout the paper: the threshold and the calibration temperature are tuned on validation probabilities produced by a training-only fit, while the coefficients are re-estimated on train+validation before the holdout is touched. Protocol B keeps the training-only coefficients, and protocol C re-anchors the threshold so that the validation alarm rate is preserved. ^†^RS denotes the uniform random-search control.

### 4.5 Comparison with conventional baselines

Panel A of Table 5 compares the optimized policies with nine reference strategies, and the comparison on the holdout window is unfavorable to optimization. Buy-and-hold SPY returns 12.00% with a Sharpe ratio of 1.118 and a maximum drawdown of −6.62%; a constant 60% exposure returns 7.19% with essentially the same Sharpe ratio and half the drawdown; a ridge-logistic model that uses every candidate predictor with a penalty selected on the validation block and a fixed 0.50 decision threshold reaches a balanced accuracy of 0.741, an area under the curve of 0.777, a 13.42% return, a Sharpe ratio of 2.280 and a maximum drawdown of −3.49%. The mean over the eleven optimized methods is 0.681 balanced accuracy and a 3.01% return, and the best method by mean return, CS, reaches 5.67%. The best single run out of the 330 runs of the eleven metaheuristics returns 12.02%, which is the order of magnitude reported in the first submission; it is a draw from the upper tail of Figure 6e rather than a property of the method that produced it. Figures 6a–d and 6f show the run-to-run distributions of balanced accuracy, sensitivity, ROC area, F1-score, and Sharpe ratio on the untouched holdout.

Table 5Conventional reference strategies on the untouched holdout window and across the five rolling-origin test blocks.

**Table:** 

Strategy	Bal. acc.	Sens.	F1	AUC	Return (%)	Sharpe	Max DD (%)	Ann. vol. (%)	Turnover	Mean exp.
Panel A. Untouched holdout window										
Buy-and-hold SPY	–	–	–	–	12.00	1.118	−6.62	12.97	0.00	1.000
Fixed exposure, *e* = 0.60	–	–	–	–	7.19	1.115	−4.00	7.78	0.40	0.600
Fixed exposure, *e* = 0.80	–	–	–	–	9.60	1.117	−5.32	10.38	0.20	0.800
Volatility targeting, σ^*^ = 10%	–	–	–	–	7.11	0.837	−6.55	10.57	3.42	0.844
Volatility targeting, σ^*^ = 15%	–	–	–	–	10.26	1.017	−6.61	12.33	1.15	0.966
VIX-threshold rule, *e*_min_ = 0	0.568	0.167	0.262	0.772	2.40	0.302	−10.32	11.78	19.00	0.938
VIX-threshold rule, *e*_min_ = 0.30	0.568	0.167	0.262	0.772	5.22	0.575	−8.93	11.89	13.30	0.956
Ridge logistic, all predictors, τ = 0.50	0.741	0.688	0.579	0.777	13.42	2.280	−3.49	6.76	15.00	0.684
Ridge logistic + manual defensive policy	0.741	0.688	0.579	0.777	6.16	0.794	−6.05	9.68	6.17	0.854
Optimized policies, mean of the eleven methods	0.678	0.577	0.496	0.752	3.00	0.514	−6.57	7.85	17.98	0.700
Best optimized mean (CS)	0.693	0.638	0.514	0.763	5.67	0.933	−5.78	7.71	16.75	0.712

**Table:** 

Test window	Stress rate	AUC of VIX level	Buy-and-hold	Fixed 0.60	Vol. target 15%	VIX rule	Ridge τ = 0.5	Ridge + manual
Panel B. Return of every reference strategy across market windows (%)								
block 1 (2018-01–2018-06)	0.705	0.842	−5.21	−3.03	−6.24	−2.20	−3.92	−6.88
block 2 (2018-06–2018-11)	0.362	0.957	−2.64	−1.52	−1.95	−2.77	4.57	−0.40
block 3 (2018-11–2019-04)	0.305	0.940	11.85	7.09	8.36	14.64	5.14	8.42
block 4 (2019-04–2019-09)	0.219	0.796	2.27	1.44	0.68	−2.49	5.10	−1.13
block 5 (2019-09–2020-02)	0.238	0.793	8.65	5.15	8.65	7.06	7.31	6.54
holdout (2019-04–2020-02)	0.230	0.772	12.00	7.19	10.26	5.22	13.42	6.16

Every reference strategy is causal and is charged the same 5 basis points per unit of absolute exposure change. The ridge-logistic baselines use all candidate predictors, a penalty selected on the validation block alone and a fixed 0.50 decision threshold; the manual policy fixes *e*_min_ = 0.20, γ = 2, σ^*^ = 15%, κ = 0.50 and *d*_cut_ = 0.10 without any search. The third column of Panel B ranks the days of each window by the current VIX level alone.

**Figure 6:**
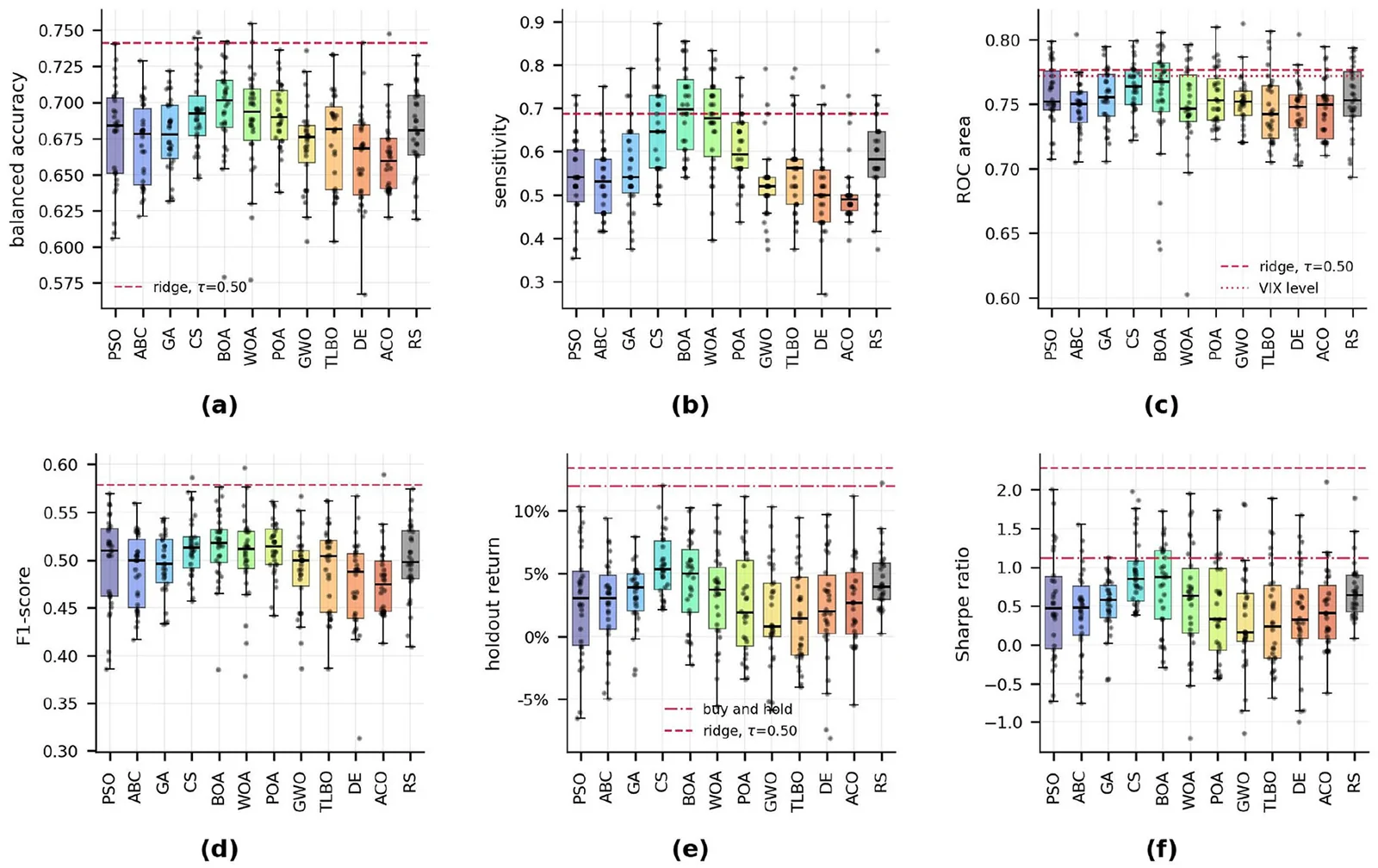
Untouched-holdout performance of the validation-selected solution of every one of the 30 independent runs: **(a)** balanced accuracy, **(b)** sensitivity, **(c)** ROC area, **(d)** F1-score, **(e)** strategy return, **(f)** Sharpe ratio. Each dot is one run. Red lines give the corresponding value of the buy-and-hold portfolio, of the ridge-logistic baseline with a fixed 0.50 threshold and of a univariate ranking by the current VIX level. RS is the uniform random-search control.

Two qualifications apply to this comparison. The holdout window is a rising market interrupted by a single volatility episode at its end, so any defensive overlay that holds average exposure near 0.70 gives up return by construction; that is a property of the window rather than a defect of the policy. Figure 7 traces the equity, drawdown and exposure paths behind these numbers, and Panel B of Table 5 shows how strongly the ranking of the reference strategies depends on the window: in the block covering the February 2018 volatility shock every one of them loses money and the ridge-logistic rule is among the worst, while in the block covering the late-2018 correction the naive VIX-threshold rule returns 15.72%. The ridge baseline is also not free of tuning, since its penalty is selected on the validation block. What the table establishes is that the joint optimization of feature subset, regularization, threshold, temperature and five policy controls bought no out-of-sample advantage over a plain regularized logistic model with a textbook threshold on this sample.

**Figure 7:**
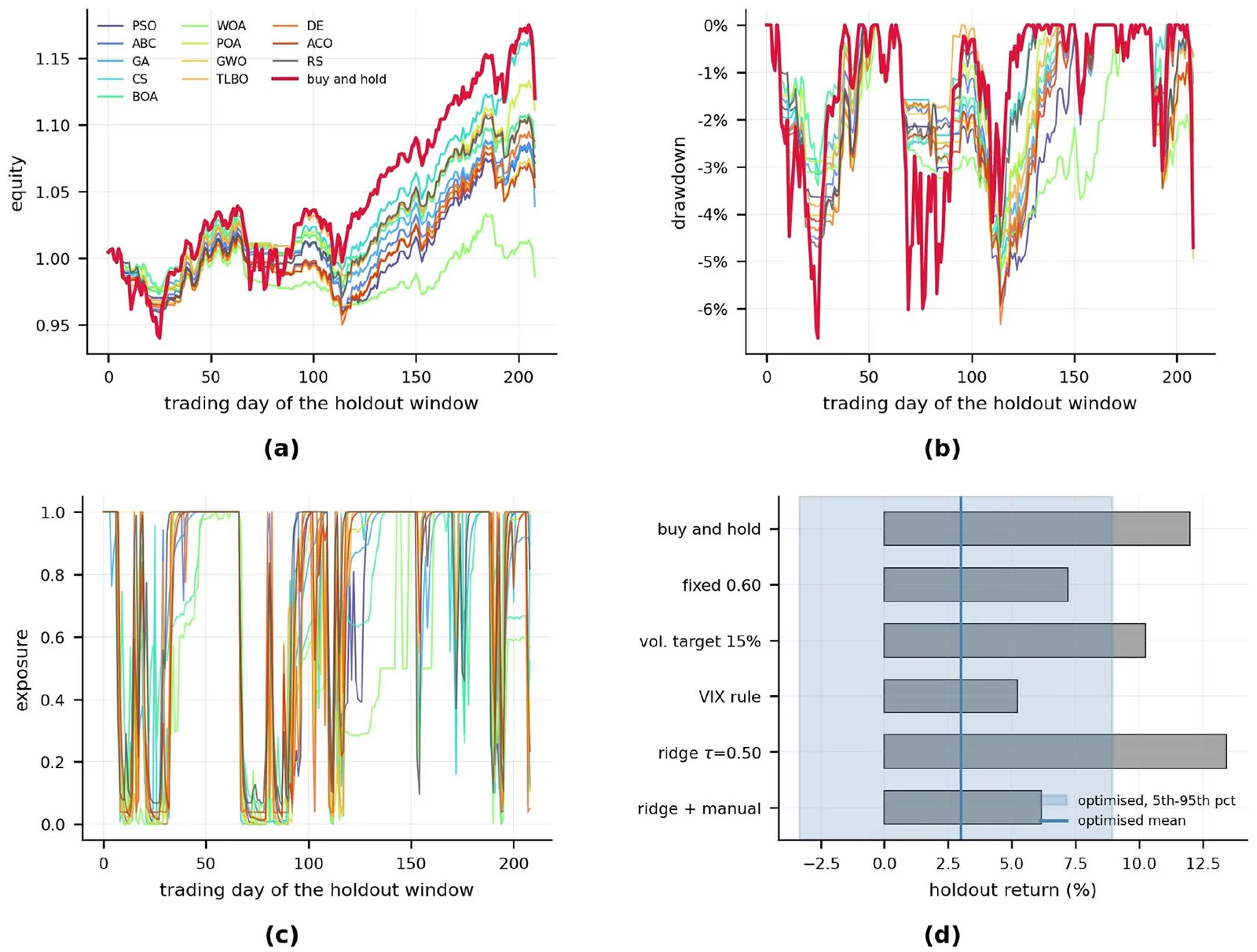
Causal defensive allocation on the untouched holdout period. **(a)** Cumulative growth of one unit of capital, **(b)** drawdown and **(c)** exposure trajectories of the representative solutions, with buy-and-hold in red. **(d)** Holdout return of every reference strategy against the mean and the 5th–95th percentile band of the optimized policies. Transaction costs of 5 basis points were charged on every exposure change.

### 4.6 Rolling-origin evaluation and stress-definition robustness

Panels A and B of Table 6 repeat the entire pipeline at five successive origins. Pooled over the 50 (origin, run) blocks, the mean balanced accuracy of the eleven metaheuristics ranges from 0.615 (GA) to 0.694 (POA) and the mean area under the curve from 0.823 to 0.842, with the random-search control at 0.850. The Friedman test rejects equality for balanced accuracy ($\chi_{F}^{2}=21.64$, *p* = 0.017) and sensitivity ($\chi_{F}^{2}=25.34$, *p* = 0.0047) but not for the area under the curve (*p* = 0.45), the return (*p* = 0.42) or the Sharpe ratio (*p* = 0.28). After Holm correction four of the 55 balanced-accuracy comparisons survive and none of the area-under-the-curve, return or Sharpe comparisons do, and no metaheuristic differs significantly from random search on any criterion. The single-holdout pattern is therefore reproduced at every origin, as Figure 8 shows. The rolling analysis also shows how much the difficulty of the task varies: stress rates across the five blocks range from 0.219 to 0.705, the pooled area under the curve is higher than on the final holdout because a block containing a persistent high-volatility regime is easier to rank, and mean returns stay between 0.23% and 1.21% over roughly five months.

Table 6Out-of-sample robustness: five rolling origins and six stress definitions.

**Table:** 

Origin	Test start	Test end	Training days	Validation days	Test days	Stress rate
Panel A. Design of the five rolling origins						
1	2018-01-26	2018-06-26	391	130	105	0.705
2	2018-06-26	2018-11-21	469	156	105	0.362
3	2018-11-21	2019-04-25	547	182	105	0.305
4	2019-04-25	2019-09-23	625	208	105	0.219
5	2019-09-23	2020-02-21	703	234	105	0.238

**Table:** 

Method	Bal. acc.	Sensitivity	AUC	F1	Return (%)	Sharpe	Features
Panel B. Performance pooled over the five origins and ten runs per origin							
PSO	0.652±.111	0.833±.212	0.840±.099	0.607±.135	0.23 ± 4.29	0.034 ± 1.565	5.9
ABC	0.672±.119	0.851±.191	0.834±.110	0.627±.126	0.47 ± 4.24	0.311 ± 1.409	6.3
GA	0.615±.112	0.851±.228	0.842±.100	0.579±.144	0.59 ± 4.29	0.287 ± 1.392	6.9
CS	0.639±.121	0.888±.171	0.842±.103	0.607±.137	1.06 ± 4.34	0.456 ± 1.587	8.4
BOA	0.647±.114	0.882±.171	0.823±.125	0.595±.132	1.21 ± 3.42	0.310 ± 1.351	4.1
WOA	0.638±.105	0.890±.156	0.832±.107	0.600±.130	0.49 ± 3.98	0.030 ± 1.670	4.3
POA	0.694±.107	0.883±.140	0.835±.109	0.631±.143	0.90 ± 4.24	0.302 ± 1.743	4.3
GWO	0.662±.116	0.859±.193	0.834±.109	0.625±.139	0.51 ± 4.73	0.204 ± 1.669	4.4
TLBO	0.691±.104	0.852±.183	0.832±.111	0.632±.141	0.60 ± 4.58	0.257 ± 1.666	4.4
DE	0.657±.124	0.837±.213	0.835±.109	0.617±.147	0.50 ± 4.55	0.128 ± 1.630	6.4
ACO	0.656±.122	0.831±.228	0.841±.106	0.608±.147	0.62 ± 4.53	0.224 ± 1.536	5.6
RS^†^	0.645±.118	0.860±.207	0.850±.099	0.602±.135	0.95 ± 4.26	0.286 ± 1.466	10.9

**Table:** 

Configuration	Predictors	Test days	Stress rate	Bal. acc.	Sensitivity	AUC	AUC of VIX level	Return (%)	Sharpe	Features
Panel C. Alternative stress definitions and the VIX predictor ablation										
VIX threshold, all predictors (published target)	28	209	0.230	0.681±.033	0.583±.115	0.756±.025	0.772	3.01 ± 3.83	0.528	5.8
VIX threshold, every VIX-derived predictor removed	22	209	0.230	0.665±.031	0.534±.097	0.734±.021	0.772	1.71 ± 4.00	0.277	5.2
Forward realized-volatility spike	28	209	0.297	0.796±.032	0.743±.085	0.880±.031	0.882	3.44 ± 3.19	0.615	6.9
Forward SPY drawdown deepening	28	209	0.287	0.708±.048	0.687±.102	0.782±.046	0.752	5.04 ± 2.77	0.864	5.6
Forward Amihud illiquidity spike	28	209	0.426	0.807±.047	0.795±.060	0.898±.014	0.873	5.04 ± 2.63	0.874	5.5
Forward composite stress-index spike	28	194	0.216	0.688±.071	0.547±.200	0.811±.030	0.739	5.73 ± 2.94	0.920	6.4

Panel B pools the 50 (origin, run) blocks; Friedman tests across the eleven metaheuristics give BA: $\chi_{F}^{2}=21.64$, *p* =.0170; Se: $\chi_{F}^{2}=25.34$, *p* =.0047; AUC: $\chi_{F}^{2}=9.85$, *p* =.4537; Return: $\chi_{F}^{2}=10.29$, *p* =.4150; Sharpe: $\chi_{F}^{2}=12.09$, *p* =.2788. After Holm correction four of the 55 balanced-accuracy comparisons remain significant, no AUC, return or Sharpe comparison does, and no metaheuristic differs significantly from the random-search control on any criterion. Every configuration of Panel C was re-optimized from the beginning with the eleven metaheuristics and ten runs each, so each row pools 110 untouched-holdout evaluations; the column “AUC of VIX level” ranks the same days by the current VIX alone. ^†^RS denotes the uniform random-search control.

**Figure 8:**
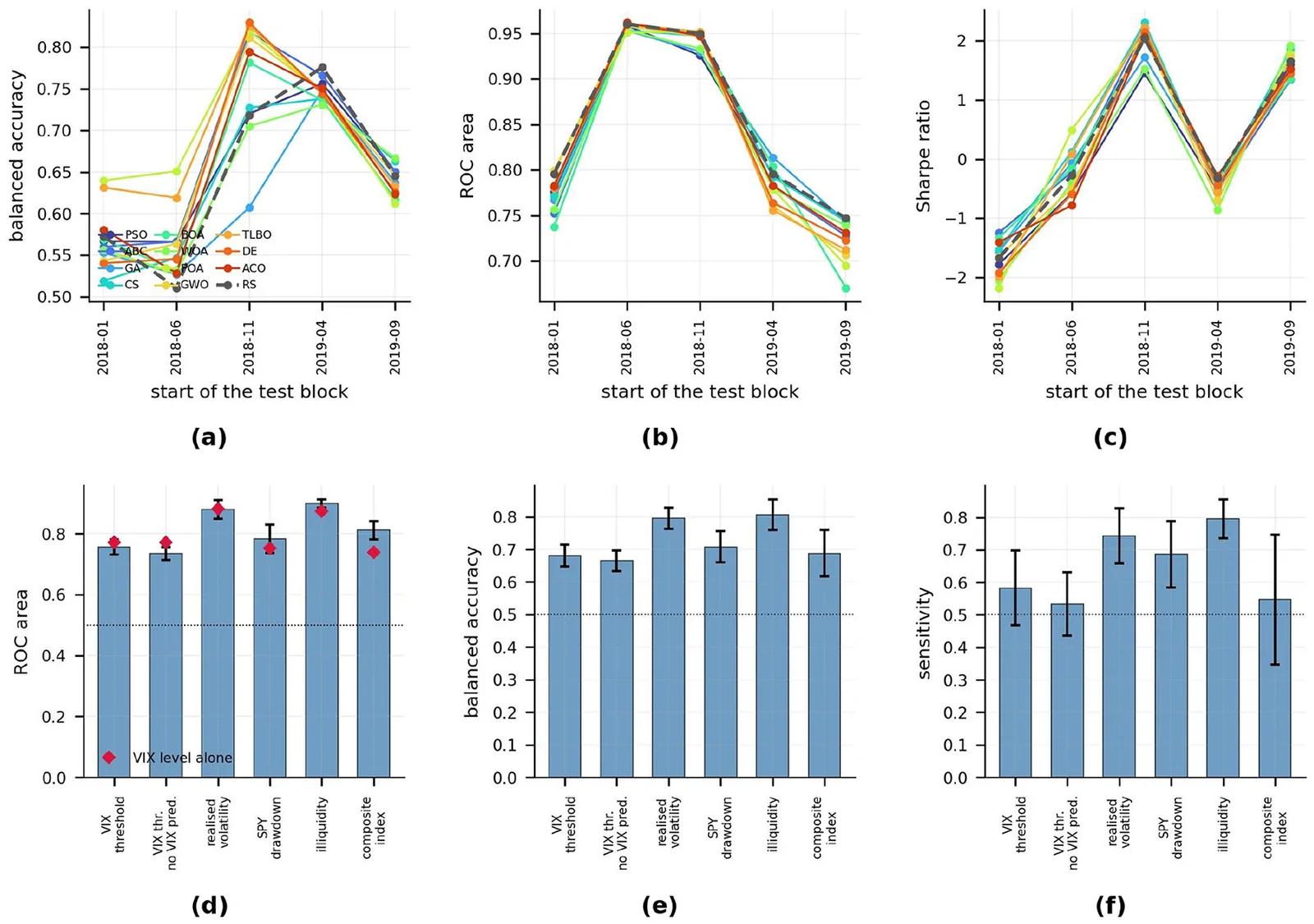
Out-of-sample robustness. Rolling-origin **(a)** balanced accuracy, **(b)** ROC area and **(c)** Sharpe ratio: each point is the mean over ten runs of one method on one untouched test block, and the dashed gray curve is the random-search control. Stress-definition robustness: **(d)** ROC area, **(e)** balanced accuracy and **(f)** sensitivity pooled over eleven optimizers and ten runs, where the red markers give the ROC area obtained by ranking the same days with the current VIX level alone.

Panel C of Table 6 reports the six label configurations. Removing every VIX-derived predictor while keeping the VIX-based label costs 0.022 points of area under the curve (0.756–0.734) and 0.016 points of balanced accuracy, so most of the discriminative content is carried by price-path, volume, drawdown and liquidity variables rather than by the VIX itself. That does not dispose of the narrowness of the target: ranking the same holdout days by the current VIX level alone gives an area under the curve of 0.772, above the 0.756 of the optimized multivariate models and above the 0.734 of the ablated models. For this label the framework does not improve on a univariate rule.

The picture changes for the other four stress definitions. With a forward realized-volatility spike the optimized models reach 0.880 ± 0.031 area under the curve against 0.882 for the VIX-level rule; with a deepening SPY drawdown, 0.782 ± 0.046 against 0.752; with an illiquidity spike, 0.898 ± 0.014 against 0.873; with a composite stress index, 0.811 ± 0.030 against 0.739. In three of these four cases the multivariate framework is clearly better than the univariate reference, and the balanced accuracies, between 0.708 and 0.807, exceed those obtained under the VIX-threshold label. The narrow behavior observed for the VIX-threshold label is therefore a property of that specific target rather than of the method.

### 4.7 Calibration, volatility regimes and recurrent predictors

Figures 9a, b separate discrimination from probability reliability. The reliability curves stay close to the diagonal in the middle of the probability range and deteriorate in the upper bins, where few holdout days fall; the expected calibration error of the representative solutions lies between 0.120 and 0.184 and the Brier scores between 0.173 and 0.207. Guo et al. (2017) showed that a discriminative model can remain poorly calibrated, and Brier (1950) established a proper scoring rule that penalizes both overconfident errors and weak resolution. The chronological probability cloud in Figure 9c shows the largest disagreement between methods around transition periods, where exposure decisions are most consequential. Figure 9d further compares balanced accuracy across the low-, medium-, and high-VIX terciles.

**Figure 9:**
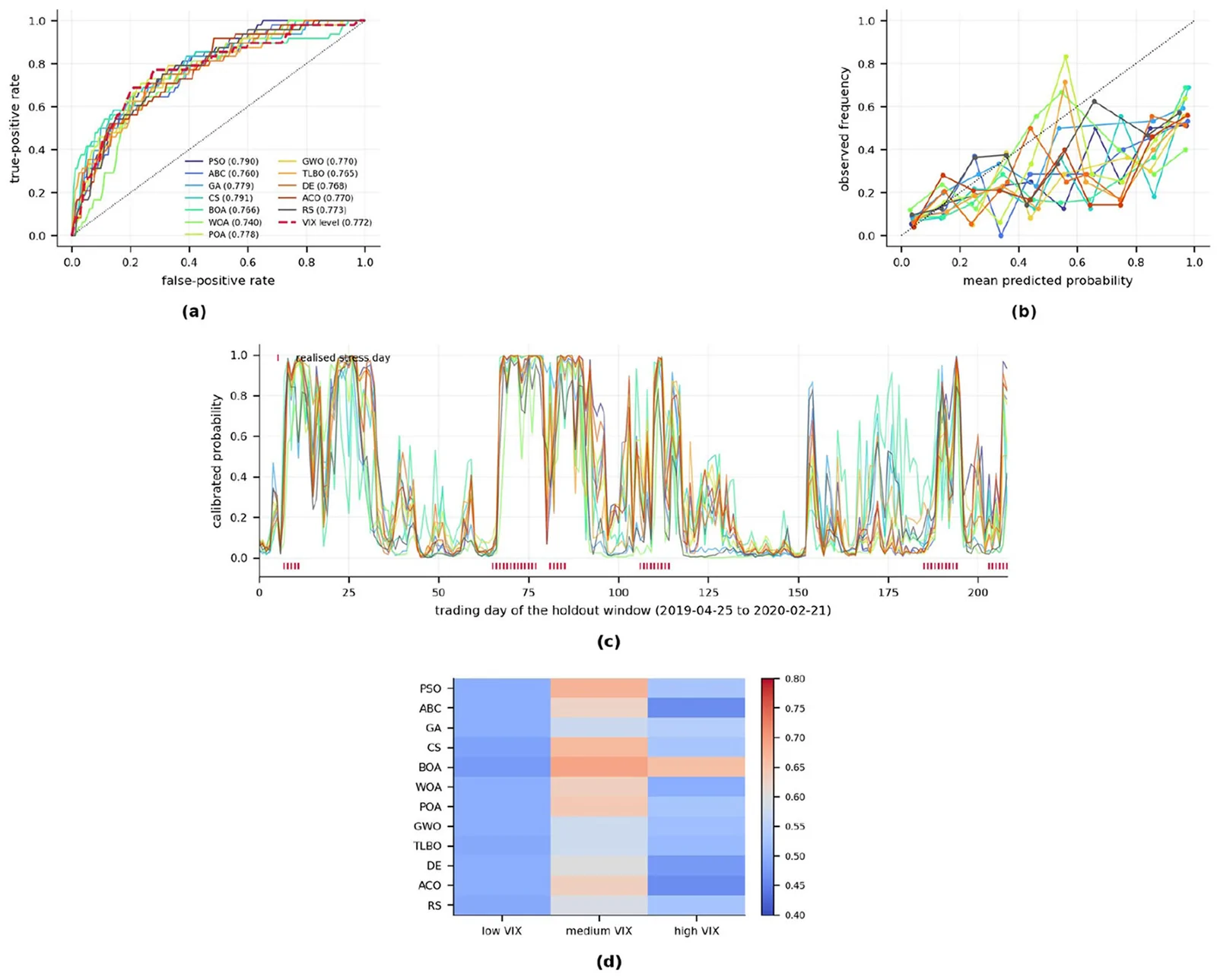
Discrimination, calibration and regime behavior on the untouched holdout. **(a)** ROC curves of the representative solutions, with the dashed red curve ranking the same days by the current VIX level alone. **(b)** Reliability curves. **(c)** Chronological cloud of calibrated stress probabilities, with realized stress days marked below the axis. **(d)** Balanced accuracy by VIX tercile.

Panel A of Table 4 shows that no method dominates every volatility regime. The low-VIX tercile of the holdout contains 71 days and only six stress events, so every method reports a sensitivity of zero and a nominal balanced accuracy near 0.5 in that regime; those cells carry no information. The medium-VIX tercile contains nine events and the high-VIX tercile 33, and only in the high-VIX tercile do sensitivities become meaningful, where they range from 0.667 to 0.909. Event counts are reported next to the metrics for that reason.

Panel B of Table 4 aggregates feature selection over all 330 optimized models rather than over eleven, which makes the recurrence pattern far more stable than in the first submission. The 60-day drawdown is retained in 73.3% of the models and the 60-day volatility in 65.8%, ahead of 20-day volume volatility at 34.2%. Persistent risk state, rather than the immediate shock, is what the search keeps. Wang and Zhou (2024) found nonlinear and threshold-like drivers of systemic stress, de Moraes Souza et al. (2024) showed that stress models benefit from several complementary explanatory variables, and Bussmann et al. (2025) demonstrated that compact financial models retain economically meaningful risk information.

## 5 Discussion

The revised evidence supports three conclusions, and only the first survives from the original version of this paper.

The multi-objective formulation is sound and informative. The seven objectives conflict, the correlation structure in Panel C of Table 3 shows that none of them is redundant, and the framework exposes trade-offs that a single weighted accuracy score would hide. The same decision vector controls feature selection, regularization, the decision threshold, the calibration temperature and five allocation parameters, which is a fully specified and reproducible formulation of that joint problem for market-level volatility regimes. Sutiene et al. (2024) argued that AI portfolio management should connect signal generation, portfolio construction, execution and evaluation; the framework operationalises that connection, and the supplementary code makes every step inspectable.

Optimization quality and decision quality are not merely different, as the first version stated, but almost unrelated on a sample of this size. The metaheuristics separate in the validation objective space, where 31 of 55 hypervolume comparisons and 39 of 55 loss comparisons survive Holm correction and where eight to ten of the eleven methods beat a random-search control with large effect sizes. They do not separate on untouched data, where no method beats random search on any criterion and where the correlation between validation search quality and holdout balanced accuracy is positive. This is the behavior that (White, 2000) and (Bailey et al., 2014) describe for repeated evaluation of trading rules on a single historical window, quantified here inside a controlled, leakage-free design. The implication for the metaheuristic literature in finance is direct: a comparison that reports only Pareto-space indicators, or only one run per method, cannot support a claim about financial usefulness.

The economic claims of the first version do not hold up against strong baselines. On the holdout window a ridge-logistic model with all predictors and a fixed 0.50 threshold outperforms every optimized configuration, and buy-and-hold outperforms the mean optimized policy. Neither result invalidates the framework: the ridge baseline uses the same causal predictors and the same cost model, and the rolling analysis shows that its advantage is window-specific, since it ranks among the worst strategies in the February 2018 block. What the baselines establish is that the gains reported in the first version came from the choice of run and from the choice of window, not from the optimization.

The robustness study in Section 4.6 restores part of the scope of the framework. Removing all VIX-derived predictors costs little, and under four alternative stress definitions the optimized multivariate models beat a univariate VIX rule in three cases, with balanced accuracies up to 0.807 for the illiquidity label. Applications of this framework should therefore prefer a composite or liquidity-based label over a VIX-threshold label.

The practical message is more modest than the one offered in the first version. GWO and ACO are the methods of choice when the goal is to cover the trade-off surface of this problem; POA and BOA settle on operating points with higher sensitivity; GA and CS are weak searchers here. None of these choices should be expected to change out-of-sample performance materially. Purata-Aldaz et al. (2025) emphasized that realistic asset-selection methods require application-specific evaluation; the present study quantifies the cost of ignoring that requirement. A practitioner who needs a defensive overlay for a single asset would be well advised to start from a simple calibrated logistic model with a fixed threshold, and to use the multi-objective machinery to understand the trade-offs rather than to extract additional return.

A final strength is reproducibility. The supplementary programs contain the data-retrieval logic, the embedded snapshot, feature engineering, objective definitions, all eleven optimizers, the random-search control, archive management, repeated-run statistics with Holm correction, the nine reference strategies, the rolling-origin driver, the alternative stress definitions, the figures and the exported tables. Every number reported above can be regenerated from them.

## 6 Limitations and future work

The most important limitation is the sample. The analysis uses 1,042 usable observations, of which 208 form the validation block on which a 28-variable feature gate and eight continuous controls are tuned. That ratio is the direct cause of the transfer failure documented in Section 4.4, and additional optimization effort cannot repair it. A design that either reduces the search space or increases the effective sample, for example through purged cross-validation with embargoes across several assets, is the natural next step.

The second limitation is the period. The data end on 28 February 2020, so the sample captures the beginning of the COVID-19 shock and none of what followed: the March 2020 collapse and recovery, the 2022 inflation and monetary-tightening regime, the 2023 regional banking stress and subsequent conditions. Every claim of regime robustness has been removed, and we do not assert that the framework would behave as reported in those periods. Extending the snapshot to the present and repeating the full protocol, including the rolling-origin and baseline analyses, is the highest-priority extension; the supplementary code accepts a longer file without modification.

Third, the label of Equation 1 is a short-horizon volatility-regime exceedance and not a broad measure of financial stress. Section 4.6 shows that the framework transfers to realized-volatility, drawdown, illiquidity and composite labels, but a genuine financial-stress study would need funding spreads, credit conditions and cross-market measures that a SPY–VIX snapshot cannot provide.

The low-VIX subset contains six positive events, which makes sensitivity estimates in that regime uninformative; event counts are now reported next to every regime metric. The ridge-logistic classifier was selected for transparency and computational comparability, and calibrated tree ensembles, temporal convolutional networks and Bayesian sequence models could be integrated while preserving the same chronological protocol. Transaction costs were modeled at 5 basis points per absolute exposure change; slippage, bid-ask spread, market impact and capacity should be linked to liquidity and trade size in a deployment study. Thirty independent runs are now used throughout, and the Holm-adjusted post-hoc analysis deferred in the first version is included; a larger study could also increase the population and iteration budget, although the evidence in Section 4.4 suggests that a stronger search would widen rather than close the gap between validation and holdout performance. The backtest is a research evaluation of decision quality and does not constitute investment advice or a claim of guaranteed profitability.

## 7 Conclusion

This study introduced a leakage-controlled multi-objective framework for short-horizon volatility-regime early warning and defensive asset allocation. Eleven established metaheuristics jointly selected indicators and tuned regularization, classification, calibration and policy controls under identical computational budgets, and the framework preserved seven separate objectives so that predictive quality, missed events, probability reliability, parsimony, temporal stability, drawdown and financial efficiency could be evaluated without concealing their conflicts.

With 30 independent runs, a random-search control, Holm-adjusted pairwise testing, nine conventional baselines, a five-origin rolling evaluation and five alternative stress definitions, the results are clear in two directions. The metaheuristics differ substantially and significantly in how well they cover the validation trade-off surface, where GWO obtains the best aggregate optimization rank and ACO the largest hypervolume and the lowest scalar validation loss. They do not differ significantly on untouched data, where no method beats uniform random search on balanced accuracy, ranking quality or risk-adjusted return, and where a ridge-logistic model with a fixed threshold matches or exceeds every optimized configuration. The framework retains value as a diagnostic instrument and as a reproducible protocol, and it performs better under realized-volatility, drawdown, illiquidity and composite stress labels than under the VIX-threshold label used here, but it does not demonstrate that metaheuristic optimization improves out-of-sample early-warning or allocation performance on a sample of this size.

The negative part of this result carries as much practical value as the positive part. Studies that compare nature-inspired optimizers on financial problems routinely report a winner on the basis of a small number of runs and one holdout window. The present experiment shows how easily that procedure manufactures a winner: the favorable single run of almost every method reaches a balanced accuracy above 0.73 and a holdout return close to 10%, while the same method averages 0.67 and below 3%. Repeated runs, a random-search control, corrected pairwise tests and conventional baselines are inexpensive, and they should accompany any claim that an optimizer improves a financial decision. The supplied MATLAB code makes the complete analysis reproducible and provides a foundation for extending the framework to other assets, longer market histories, alternative stress definitions and more advanced calibrated classifiers.

## Data Availability

Publicly available datasets were analyzed in this study. This data can be found here: Direct data link: https://raw.githubusercontent.com/hackingthemarkets/vix-strategy/refs/heads/master/spy_vix.csv Repository name: GitHub repository: hackingthemarkets/vix-strategy; Repository link: https://github.com/hackingthemarkets/vix-strategy.
